# A Compendium of the Most Promising Synthesized Organic Compounds against Several *Fusarium oxysporum* Species: Synthesis, Antifungal Activity, and Perspectives

**DOI:** 10.3390/molecules26133997

**Published:** 2021-06-30

**Authors:** Paola Borrego-Muñoz, Felipe Ospina, Diego Quiroga

**Affiliations:** Bioorganic Chemistry Laboratory, Facultad de Ciencias Básicas y Aplicadas, Campus Nueva Granada, Universidad Militar, Nueva Granada, Cajicá 250247, Colombia; u7700101@unimilitar.edu.co (P.B.-M.); felipe.ospina@udea.edu.co (F.O.)

**Keywords:** vascular wilt, antifungal agent, *FOX*, IC_50_, MIC, organic synthesis

## Abstract

Vascular wilt caused by *F. oxysporum* (*FOX*) is one of the main limitations of producing several agricultural products worldwide, causing economic losses between 40% and 100%. Various methods have been developed to control this phytopathogen, such as the cultural, biological, and chemical controls, the latter being the most widely used in the agricultural sector. The treatment of this fungus through systemic fungicides, although practical, brings problems because the agrochemical agents used have shown mutagenic effects on the fungus, increasing the pathogen’s resistance. The design and the synthesis of novel synthetic antifungal agents used against *FOX* have been broadly studied in recent years. This review article presents a compendium of the synthetic methodologies during the last ten years as promissory, which can be used to afford novel and potential agrochemical agents. The revision is addressed from the structural core of the most active synthetic compounds against *FOX*. The synthetic methodologies implemented strategies based on cyclo condensation reactions, radical cyclization, electrocyclic closures, and carbon–carbon couplings by metal–organic catalysis. This revision contributes significantly to the organic chemistry, supplying novel alternatives for the use of more effective agrochemical agents against *F. oxysporum*.

## 1. Introduction

The genus *Fusarium* is considered one of the most adaptable and versatile in Eumycota. One of its most economically important species is *FOX*, an invasive phytopathogen responsible for vascular wilt and cortical rot in more than a hundred crops of commercial interest. *FOX* is responsible for a large percentage of economic losses in the agricultural sector worldwide. These phytopathogens have host specialization capacity and high virulence, becoming a broad complex of *FOX* species with a high probability of new pathogens [1]. This complexity of species has gained considerable attention in the scientific community in recent years. The Molecular Plant Pathology Journal has included *FOX* among the “Top 10” of the phytopathogenic fungi based on its scientific and economic importance [2].

*FOX* complexes are distributed worldwide. The pathogen accumulates in sufficient inoculums, and then a susceptible cultivar is planted. The symptoms of the plant can be evidenced through chlorotic flakes, which undergo curvature and lose structural stability. The plant eventually wilts which can be evidenced by acquiring a yellowish-brown color while the vascular system changes color from light yellow to brick red. Most of the forms in *FOX* complexes exist as chlamydospores, which remain latent in the host’s tissue and soil until they stimulate germination, with exudates from the roots responsible for their germination. Various pathogenic forms of *FOX* can enter the host root through wounds or directly through the root tips [3,4].

The impact of the *FOX* species complex has generated millions of economic losses. In banana crops [5,6,7], the losses caused by the TR4 race of *F. oxysporum f.* sp. were estimated at USD 2000 million during the “Gros Michel” era. TR4 is currently expected to cause even more significant losses eventually: in Latin America, from 1995, the disease of Panama was reported in most banana producing regions, except Papua New Guinea, the South Pacific Islands, and some of the countries bordering the Mediterranean; in Indonesia, Taiwan, Malaysia, China, and the Philippines, losses of around USD 253 million were estimated between 2011 and 2013 [8,9]. In tomato, the only vegetable crop cultivated globally, vital for the daily diet and consumed as freshly unprocessed fruits, millions of losses have been reported due to vascular wilt caused by *FOX* [10]. A reduction in 50% of Africa production has been reported for oil palm due to vascular wilt caused by *FOX* [3,11].

Moreover, huge losses have been reported in melon crops caused by *FOX*, carnation, and chrysanthemum flowers and cotton [12]. In countries such as the USA, China, and India, which provide approximately 35% of the total fiber use globally, *FOX f.* sp. *vasinfectum* diseases caused losses between 0.4 and 1.0% [13]. In Colombia, the cape gooseberry (*Physalis peruviana* L.) production suffered significantly from 2009–2013 due to the *FOX* complexes’ proliferation [14].

In addition to the enormous impact caused in agriculture and the economy, it is known that the toxins secreted by *FOX* complexes can cause alteration in animals and humans health [15]. The disease caused by eating food with mycotoxins is called mycotoxicosis. Several hundred compounds have been described as toxic or potentially toxic secondary metabolites of *FOX* complexes with high toxicity, demonstrated in bioassays or feeding studies. Some mycotoxins, such as enniatins, fusaric acids—inducers of cell death in tomato plants—and moniliformin, have been linked to toxicosis in humans or livestock animals immunocompromised infections in humans [16,17]. A risk factor for fusariosis can occur in immunocompetent patients due to tissue degradation caused by trauma, severe burns, or foreign bodies in the body. Infections in humans with *FOX* complexes can cause local, focally invasive, or disseminated diseases. Skin lesions can be seen in approximately 75% of cases and are usually located on the trunk and extremities, causing keratitis and onychomycosis. *Fusarium* can also affect deep skin ulcers, third-degree burns, and surgical wounds. In other cases, the infection remains localized in the immunocompetent or immunosuppressed host, causing manifestations such as septic arthritis, skin infection, central line sepsis, endophthalmitis, osteomyelitis, cystitis, and brain abscess [18,19,20,21].

To perform effective control of *FOX* complexes is one of the most difficult to achieve. Therefore, various methods have been developed, such as cultural, biological, botanical, genetic, and chemical controls. Some fungi have been evaluated more frequently in biological control methodologies than bacteria, such as *Trichoderma* (53% of fungi) [22,23]. Non-pathogenic *Fusarium* species (23%) and *Penicillium* (10%) are other used microorganisms. Concerning the bacteria uses, *Pseudomonas* (44%), followed by *Bacillus* (13%) and *Streptomyces* (9%), has been broadly employed [24]. Despite the advantages of biological control, microorganisms and botanicals can show a low range of efficiency levels. Nearly a third of the tested microorganisms have been shown to reduce the disease by only 10% to 40%. As for botanicals, most of the reported studies were performed only in vitro tests. These considerations lead to the question of the level of efficiency required to consider the marketing of a biological control product and restrict their field of application [12].

Genetic investigations on *FOX* complexes have shown promissory results towards phytopathogen control. Recent comparative analysis of the *FOX* genomes provided information on the genome’s organization and the genomic region that governs pathogenicity, revealing that each specialist form’s effector repertoire probably determines the specificity of the host [25]. Through comparative analysis, pathogenicity-related chromosomes have been identified in *FOX* that contain genes for host-specific virulence [26]. On the other hand, genetic engineering advances have been possible using new resistant genotypes of several plants through gene editing and high-performance phenotyping. These success factors depend on the genotype and are related to the level of resistance to *FOX* complexes. Strategies for integrated management should consider increasing plant defenses and suppressing *FOX* complexes in the soil once the disease is present [9,27,28]. However, the productivity and market acceptance for somaclones is lower, and there is low and expensive productivity, especially in small markets for other cultivars. Due to these considerations, pests’ chemical control through chemical products (agrochemicals as pesticides) is the most profitable and effective alternative for crops in large areas.

Fungicides with a benzimidazole group in their structure, such as benomyl, carbendazim, and thiabendazole, have demonstrated their capacity to control *FOX* complexes in vitro and under greenhouse conditions. Other agrochemicals such as cyproconazole, propiconazole, and prochloraz showed reduced *Fusarium* wilt disease of about 80% in banana plants. Soil fumigated with methyl bromide effectively reduced Panama disease in South Africa for a few months. However, the fungus was able to repopulate these soils and infect susceptible banana plants. Phosphonate compounds are potent against this phytopathogen as they reduce fungal growth under in vitro conditions. Carbendazim injections into the corm tissue of Rasthali cultivars in India provided short-term tolerance, but the results were erratic with the same treatments in other parts of the world. The disinfection processes of contaminated machinery and agricultural implements used sodium hypochlorite and detergents effectively against conidia and chlamydospores of *F. oxysporum f.* sp. However, they do not apply to large plantations, and it is known that they cause some environmental risks and even harm farmers [9,29,30]. In some countries, prochloraz and azoxystrobin replaced benomyl to control various ornamental *Fusarium* wilts and bulb cultures. However, prochloraz has never been registered for ornamental plants in the USA. The price of azoxystrobin has limited its use in many bulb crops [5].

Despite this, several measures must be considered when using agrochemicals since the side effects can be even more harmful than the pathogen. These effects only appear when the amount of pesticide in the body is more significant than what it can eliminate, so it accumulates and reaches the toxic level [31]. Frequent use of pesticides can harden or stunt cultivars of a species. Combined with the incomplete effectiveness of chemical treatments against *Fusarium* wilt, these considerations have made chemical control disappointing for farmers and the productive sector. Many times, “cocktails” of the mentioned agrochemicals are used, enhancing their effects and causing chemical alterations that originate new chemical substances [32]. The use of these mixtures and their storage without due control has caused severe health disorders for producers and their families, mainly at the reproduction level. The increased risks during pesticide application often result from a lack of information, knowledge, awareness, and inadequate supervision during the application and sale of highly toxic products. Due to this, several alternatives have been sought that allow chemical pest control to be carried out with minimal impact. Specifically, to reduce fungi’s presence in the post-harvest stage, studies have been carried out that contemplate the use of extracts from resistant plants [33]. This alternative is impressive considering that the secondary metabolites with biological activity are the terpenoid type, phenolic compounds, phenylpropanoids, stilbenes, alkaloids, saponins, and heterocyclic compounds [34], whose advantage corresponds to their rapid degradation in the soil. Therefore, new agrochemical agents’ synthesis continues to be a profitable one and still to be explored. In this review, we present a compendium of organic compounds active against different species of *FOX*, their synthesis methods, and some recommendations and perspectives of the authors, formulated during the process of consolidation of the information to redirect the search for new molecules active against *FOX* systematically and rationally.

## 2. Compendium of the Organic Molecules with the Highest Reported Antifungal Activity against *FOX* Species

A bibliographic review was carried out in a time window between 2010 and 2020 to establish the organic molecules with the most significant biological activity against *FOX*. It was evidenced that the expression of the antifungal activity in the manuscripts differs concerning the units used or the property defined for this purpose, for example, half-maximal inhibitory concentration (IC_50_), half-maximal effective concentration (EC_50_), minimum inhibitory concentration (MIC), or percentage of inhibition (%) at a specific concentration. This review took the IC_50_, EC_50_, or MIC value expressed in micromolar as a criterion to select the interest reports. We discarded those reports where the activity was only reported as a percentage of inhibition. They were not conclusive, or merely the employed method did not provide definitive quantitative information for the corresponding molecules. Thus, a compendium of active organic molecules against various *FOX* was established in this review, presented in Table 1. The molecules were divided according to their structural core (scaffold), presumed to be a “pharmacophore” behavior. The antifungal activity of each group’s most active molecule is presented, indicating the value reported for the activity and its mathematical conversion to micromolar. This value is included in parentheses (Table S1). The order of the compounds in Table 1 was defined as decreasing the antifungal activity.

## 3. Synthetic Methods for Highly Active Compounds against *FOX* Species

### 3.1. Open-Chain and Homoaromatic Core Molecules

**Scheme 1:**
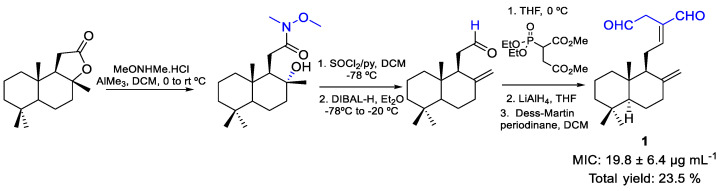
Synthesis of active molecule **1**.

Labdan-type diterpenes have reported a broad spectrum of different biological activities, including antifungal activity [35]. González et al. (2010) reported the synthesis of (+)-labdadienedial and several derivatives with yield percentages of around 90% (Scheme 1). Gonzalez et al. (2019) performed some derivatizations, such as introducing an oxygenated fragment (such as a methyl ester, alcohol, an acid, or an ester) into the lipophilic backbone of labdane (Scheme 1) [36]. These derivatives were evaluated on *FOX*, showing that compound **1** was the most active against this phytopathogen with a MIC value of 19.8 ± 6.4 µg/mL.

**Scheme 2:**
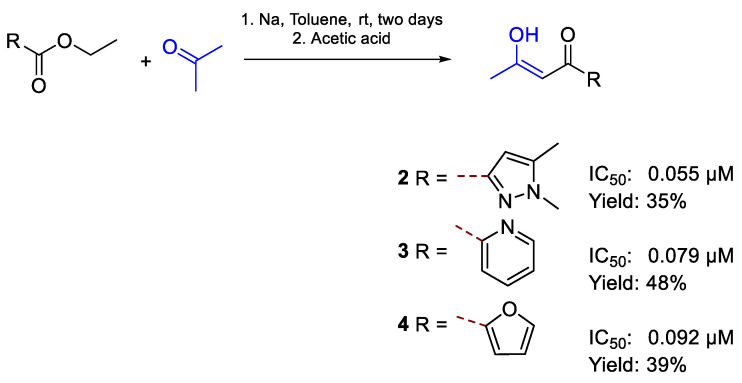
Synthesis of active molecules **2**–**4**.

Curcumin analogs have been broadly studied in recent years because it is presumed that the presence of β-keto-enol core acts as a pharmacophore, which confers antioxidant capacity. Their presence is presumably responsible for various biological activities such as anti-HIV, anti-tumor, and anti-inflammatory. Furthermore, this core has been widely used to design multiple molecules, such as some calix[4]arene derivatives containing residues linked to rings of the tetrazole and triazole type, which have shown good behavior anti-HCV agents [37]. Radi et al. (2015) [38] synthesized several hybrid derivatives bearing the β-keto-enol functionality and a heterocyclic fragment using one-pot sodium metal-mediated in situ condensation between ketone derivatives and ethyl heterocycle-2-carboxylates (Scheme 2). The products were obtained under mild conditions but low yields (22–48%), identified as β-keto-enol derivatives exclusively in their tautomeric enol form. All synthesized compounds were tested against *FOX f.* sp albedinis, and the results were reported as IC_50_ in micromolar. Compounds **2**–**4** showed the highest antifungal activities, suggesting connectivity between the β-keto-enol pharmacophore and the heterocyclic ring, in a position adjacent to the ring system’s heteroatom, enhances the antifungal activity against *FOX f.* sp. albedinis.

**Scheme 3:**
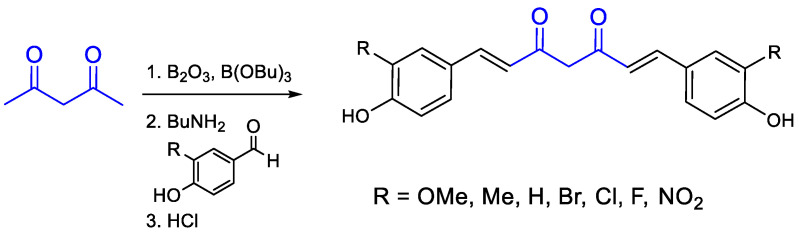
Synthesis of curcumin analogs.

Recently, a series of curcumin analogs with high potential as anti-inflammatory agents were synthesized using a condensation reaction of 1,3-dicarbonyl compounds and aromatic aldehydes in the presence of B_2_O_3_/B(OBu)_3_ mixtures with excellent yields (Scheme 3). These analogs showed minor anti-inflammatory effects in vitro and in vivo compared to curcumin. The results agree with other studies, which revealed that bisdimethoxycurcumin had reduced anti-inflammatory and anti-cancer effects [39]. This methodology can be considered an alternative to afford novel antifungal agents against *FOX*, starting from the antecedent of Radi et al.

**Scheme 4:**
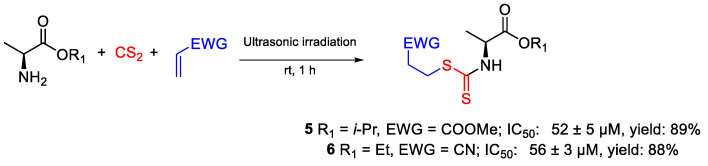
Synthesis of active molecules **5** and **6**.

Alkyl dithiocarbamates are among the open-chain molecules that can be biologically active. Quiroga et al. (2019) have obtained a series of *N,S*-dialkyl dithiocarbamates starting from 2-amino acids [40]. The synthetic protocol involves the previous derivatization of the 2-aminoacid towards alkyl 2-aminoesters using the Li and Sha methodology [41]. Then, ultrasound-assisted three-component one-pot reactions were performed between alkyl 2-amino esters, carbon disulfide, and each one of the following Michael acceptors: acrylonitrile, mesityl oxide, and methyl acrylate (Scheme 4). *N*,*S*-dialkyl dithiocarbamates were successfully obtained with moderate to high yields. The synthesized compounds were evaluated against *FOX* spp. Furthermore, the results were expressed as IC_50_. Compounds **5** and **6** showed high antifungal activity showing IC_50_ values of 56 and 52 micromolar. QSAR-3D studies allowed to establish a relationship between the structure and antifungal activity. The presence of a smaller side chain in alkyl 2-aminoester moiety (such as L-alanine) and a cyanoethyl substitution as an electron-withdrawing group enhance the antifungal activity, which can be rationalized by the efficient absorption ability through the wall of fungal hyphae, enhancing antifungal activity.

**Scheme 5:**
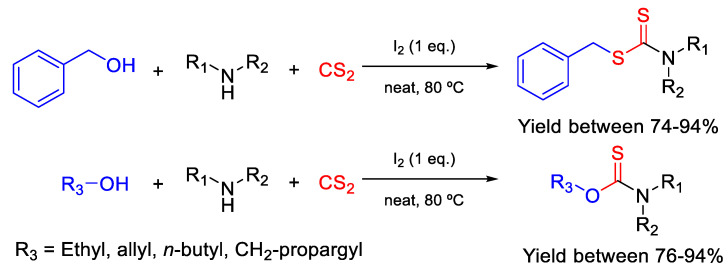
Synthesis of *S*-benzylic dithiocarbamate esters and *O*-ethyl thiocarbamates.

Although Quiroga et al. offer an adequate methodology to obtain alkyl dithiocarbamates, the performance in all cases is not excellent and is challenging, primarily due to the type of precursor used: Michael acceptors. Dutta and Saha (2020) recently developed a solvent-free synthetic methodology to afford *S*-benzylic dithiocarbamate esters via the iodine mediated direct C-S coupling benzylic alcohols with dithiocarbamate anions generated in-situ (Scheme 5) [42]. Although this methodology widens the structural variability according to the type of precursors used, the course of reaction changes in *S*-ethylation of the dithiocarbamate anion using alcohols affording *O*-ethyl thiocarbamates under the identical reaction condition. Allyl alcohol, *n*-butyl alcohol, and propargyl alcohol also reacted similarly to produce the *O*-thiocarbamate compounds with good yields (>76%).

**Scheme 6:**
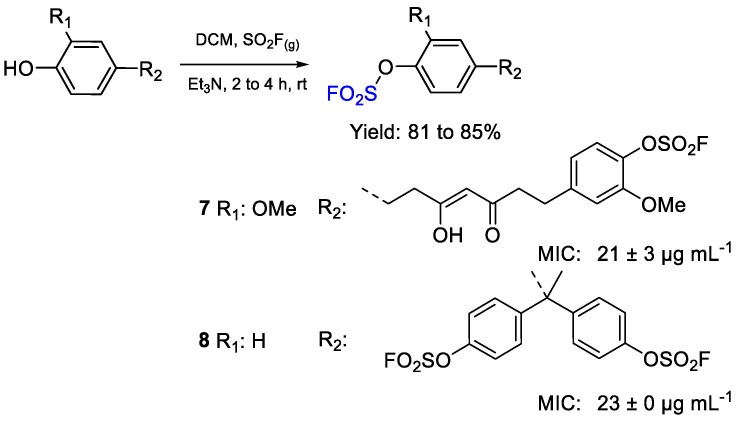
Synthesis of active molecules **7** and **8**.

Aryl fluorosulfate type compounds are typically obtained using sulfuryl fluoride (SO_2_F_2_) as a precursor, a gaseous substance with low toxicity. These compounds have been used as precursors of many applications in chemical biology, molecular pharmacology, and medical chemistry [43]. Recently, Ravindar et al. (2018) [44], inspired by the particular characteristics of these analogs, carried out the synthesis of a series of aryl fluorosulphates using the reaction of phenols against SO_2_F_2_ following the protocol described by Sharpless [45], which is carried out in the presence of a base such as triethylamine (TEA). It is known that, depending on the nature of the R substituent, the -OSO_2_F group can be an excellent leaving group, making these types of compounds interesting synthetic precursors. Concerning this reaction, aqueous–organic biphasic conditions suppress the competitive fluorosulfonation of several nucleophilic groups than phenols in differently functionalized molecules, which evidenced chemoselectivity towards phenolic hydroxyl groups. Sterically hindered substrates perform better using phenolate anions as nucleophiles in the reaction against SO_2_F_2_. Ravindar obtained all the derivatives with yields from good to excellent (Scheme 6). Furthermore, the antifungal activity in vitro against *FOX* was evaluated for all synthetic products employing the agar well diffusion method. The results of antifungal activity expressed as MIC in µg/mL (compounds **7** and **8** were highly active against *FOX*s, Table 1) suggested that the presence of methoxy and –OSO_2_F groups in the core phenyl group can enhance antifungal activity.

**Scheme 7:**
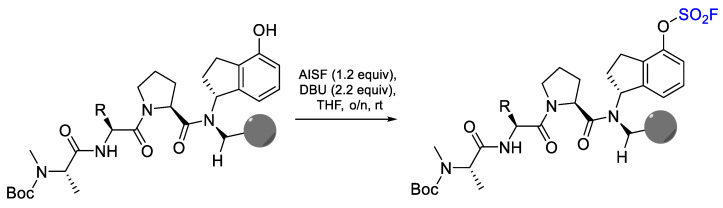
Synthesis of functionalized apoptosis protein antagonists (IAP).

Recently, Baggio et al. (2019) prepared a series of apoptosis protein antagonists (IAP), including arylfluorosulfate groups on various peptides. His synthetic protocol involved the use of 4-[(acetylamino)phenyl]imidodisulfuryl difluoride reagent (AISF) in the presence of 1,8-diazabicyclo[5.4.0]undec-7-ene (DBU) in tetrahydrofuran (THF), carrying carried out derivatization on a phenolic group bound in a peptide matrix, under mild reaction conditions (Scheme 7) [46].

**Scheme 8:**
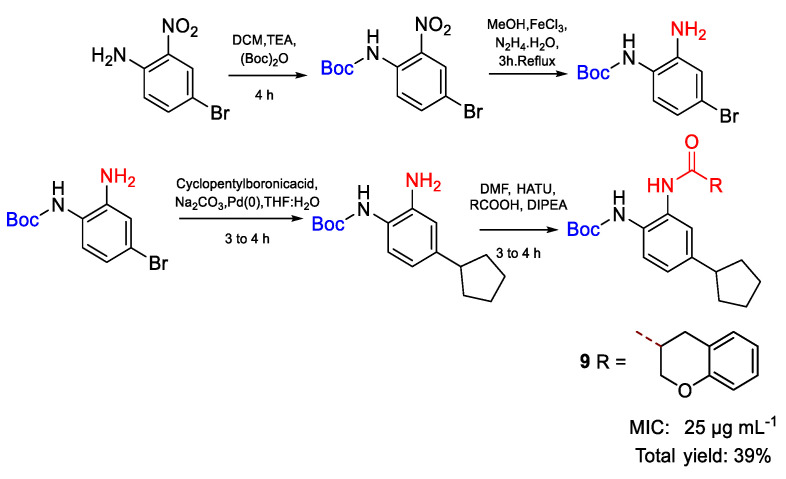
Synthesis of active molecule **9**.

Derivatives of the carbamate-type are associated with a broad spectrum of biological activities. Besides, they are considered versatile molecules due to their behavior as intermediaries in synthesizing therapeutic agents. The presence of a 5-chloroquinoline heterocyclic system in heteroarylphenyl carbamates evidenced high yield and improved antiangiogenic activity. Pochampally et al. (2014) synthesized several substituted amide derivatives from phenyl carbamates biologically actives (Scheme 8) [47]. A linear synthesis strategy starting from 2-nitro-4-bromoaniline was established, thus, after protection of amino group with Boc, the nitro group was reduced to obtain *tert*-butyl-2-nitro-4-bromophenylcarbamate. The Suzuki coupling reaction with cyclopentylboronic acid in the presence of sodium carbonate, Pd (0), and THF: H_2_O mixture afforded *tert*-butyl 2-amino-4-cyclopentyl phenylcarbamate, which finally reacted with different carboxylic acids in the presence of HATU/DMF and DIPEA (*N*,*N*-diisopropylethylamine) (63–71% of yield). The antifungal activity tests against *FOX* showed that the most active compounds in the series were **9**, which possess a heterocyclic 3,4-dihydro-2*H*-1-benzopyran system that improves the biological activity.

**Scheme 9:**
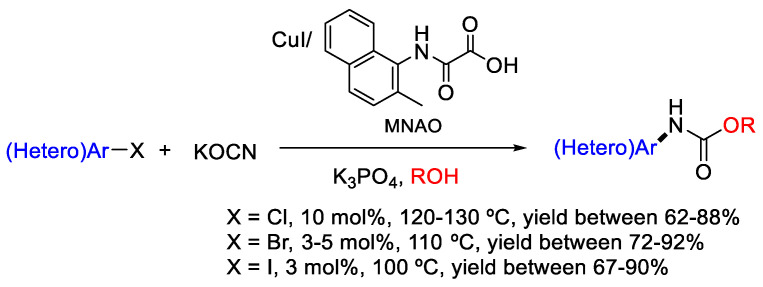
Synthesis of *N*-(hetero)aryl carbamates reported by Kumar and Ma.

The carbamate group formation step can limit the overall yield of the synthesis of several compounds. Some protocols have been reported in the literature to overcome this difficulty, employing metal-catalyzed reactions affording aryl and heteroarylcarbamates in higher yields. Kumar and Ma (2018) developed a route for the synthesis of *N*-(hetero)aryl carbamates by cross-coupling between (hetero)aryl chlorides with potassium cyanate catalyzed by CuI and 2-((2-methylnaphthalene-1-yl)amino)-2-oxoacetic acid (MNAO) in several alcohols [48]. This method uses various substrates generated *in situ* to produce various *N*-(hetero)aryl carbamates in good to excellent yields (Scheme 9). This methodology allows direct access to a wide range of aryl *N*-(hetero)carbamates from less reactive and less expensive (hetero)aryl chlorides. The ready availability of starting materials coupled with this protocol’s efficiency should make it a valuable complement to existing methods for the synthesis of *N*-(hetero)aryl carbamates.

**Scheme 10:**
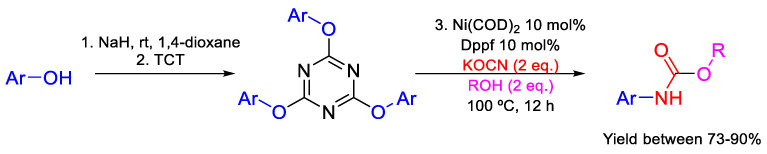
Synthesis of *N*-(hetero)aryl carbamates reported by Inaloo.

Inaloo et al. (2020) have reported a methodology for the synthesis of *N*-(hetero)aryl carbamates that use a domino reaction in a single vessel from alcohols and (hetero)aryl isocyanates produced *in situ* in the presence of a catalyst of nickel [49]. The phenolic C-O bond is activated in this protocol by phenol’s reaction with cyanuric chloride (2,4,6-trichloro-1,3,5-triazine, TCT). This strategy provides practical access to *N*-(hetero)aryl carbamates in good to excellent yields (Scheme 10) with high functional group compatibility.

**Scheme 11:**
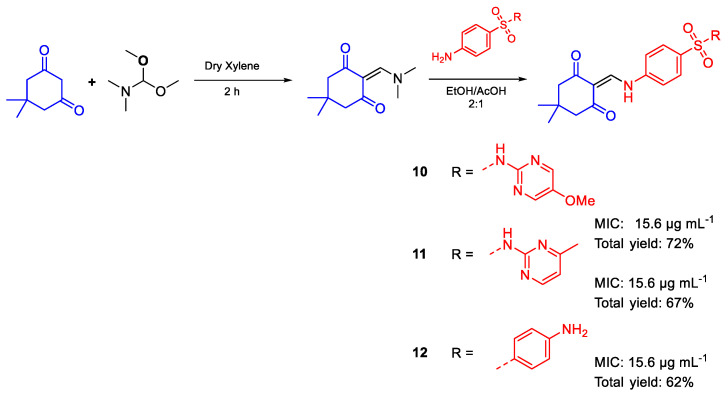
Synthesis of active molecules **10**–**12**.

Sulfa drugs are widely known to be the first antimicrobial agents discovered, effective against pyogenic bacterial infections. Ghorab et al. (2017) carried out the synthesis of some novel dimedone derivatives with sulfonamide groups [50]. This synthetic methodology involves the reaction of 5,5-dimethylcyclohexane-1,3-dione (dimedone) with dimethylformamide-dimethylacetal (DMF-DMA) in dry xylene under reflux conditions, which allowed to obtain 2-((dimethylamino)methylene)-5,5-dimethylcyclohexane-1,3-dione (yield > 94%). Nucleophilic addition reactions with 4-(phenylsulfonyl)anilines or 4-(heteroarylsulfonyl)anilines allowed to obtain several sulfa drugs (Scheme 11). The compounds with the highest antifungal activity against *FOX* were **10**–**12**. These compounds possess a six-member aromatic or heteroaromatic ring in the sulfonamide group, which can be considered potential pharmacophores.

**Scheme 12:**
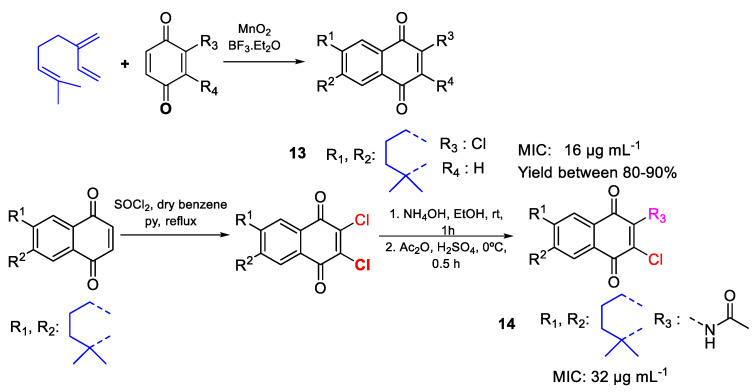
Synthesis of active molecules **13** and **14**.

Most natural naphthoquinones are previously evaluated as antifungal agents. Several studies have reported the antifungal activity of natural and synthetic quinones, mainly substituted 1,4-naphthoquinones, or compounds with structures containing various heterocyclic systems fused to the quinone fragment. Castro et al. (2013) studied the influence of a prenyl substitution in the benzene ring of 1,4-naphthoquinones and subsequent cyclization to the corresponding anthracene-1,4-dione derivatives antifungal activity this type of compounds against several fungi, among them *FOX* [51]. The synthetic strategy uses prenylquinones obtained in an initial Diels-Alder condensation catalyzed between myrcene and *p*-benzoquinone (or 2,5-dichlorobenzoquinone) the presence of BF_3_.OEt_2_, followed by oxidation of the resulting cycloadducts with MnO_2_ (Scheme 12). The absence or presence of small amounts of the catalyst during oxidation leads to regioselectivity results in these reactions. Compound **13** was obtained using microwave irradiation in the presence of SiO_2_ as solid support, followed by oxidation with MnO_2_, to produce a 1:4 mixture of the two possible position isomers. Compound **14** was obtained from dichloroquinones prepared respectively from the 1,4-naphthoquinone derivative by treatment with SOCl_2_. A substitution reaction using ethanolic NH_4_OH was then performed, followed by acetylation and chromatographic separation of both regioisomers. Compounds **13** and **14** showed good antifungal activity against *FOX* (ATCC 4811216): 16 µg mL^−1^ and 32 µg mL^−1^, respectively.

**Scheme 13:**
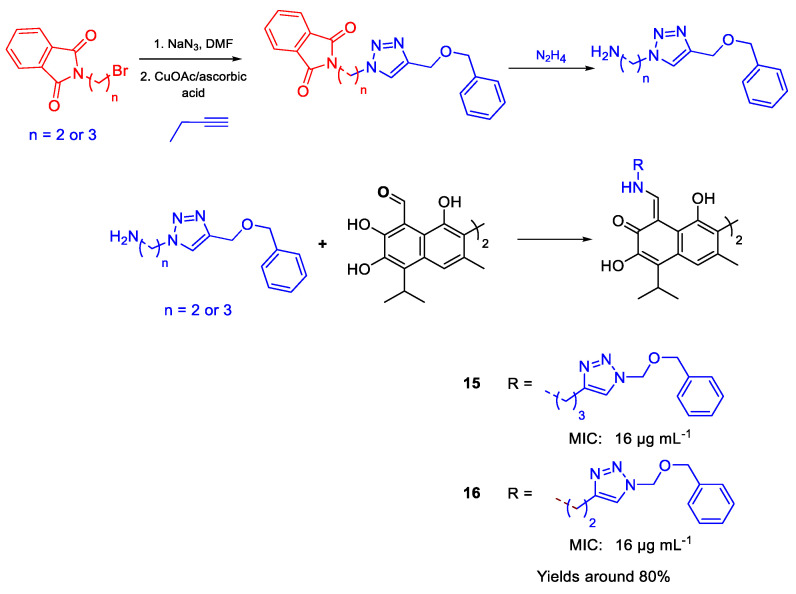
Synthesis of active molecules **15** and **16**.

Pyta et al. (2016) performed some modifications in the synthesis of new gossypol-triazole conjugates and gave rise to new compounds that showed potential antifungal activity [52]. Gossypol was subjected to functionalized primary amines’ reactions, obtaining Schiff bases in excellent yields (Scheme 13). However, the initially proposed methodology was modified, such that the phthalimide *N*-alkyl bromides were converted into phthalimide *N*-alkylazides. Dipolar cycloaddition using various alkynes in the presence of CuOAc as a catalyst allowed access to 1,2,3-triazole rings. The subsequent reaction with hydrazine produced a series of amine-triazole intermediates with moderate or good yields (60–75%), which were finally condensed with gossypol to obtain gossypol triazole conjugates functionalized with aliphatic chains and benzyloxy groups, with good yields (around 80%). The synthesized compounds showed the highest activity against *Fusarium* spp. strains, except *F. acuminatum.* The most exciting results were obtained for *Fusarium* spp. strains isolated from plants (cabbage and tomatoes), with **15** and **16**, were identified as the most active compounds (MIC = 16 μg/mL), suggesting that derivatives containing triazole-benzyloxy moieties are comparable to miconazole, a widely used commercial antifungal agent.

**Scheme 14:**
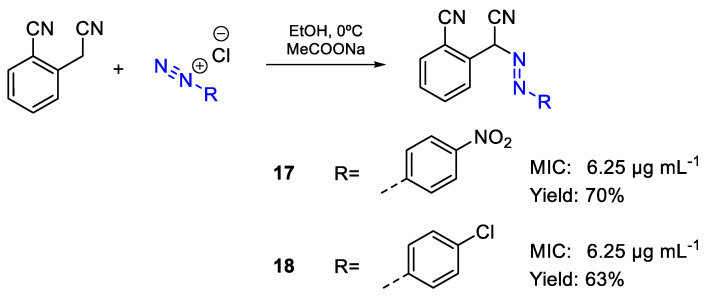
Synthesis of active molecules **17** and **18**.

Fadda et al. (2013) synthesized several benzo and naphtonitrile derivatives starting from 2-(cyanomethyl)benzonitrile. The reaction of this precursor and different diazonium salts allowed the obtain of aryldiazenyl products (Scheme 14), used as intermediaries in the synthetic protocol. However, aryldiazenyl derivatives were also evaluated to determine their antifungal activity in vitro level against *F. oxysporum*, using the agar diffusion method. The compounds **17** and **18** showed inhibition (MIC 6.25 µg/mL); however, with lower activity than cycloheximide (MIC 3.125 µg/mL), used as a positive control. Its biological activity was correlated with the low density of electrons in aromatic ring systems [53].

### 3.2. Heterocyclic Ring Core Molecules

#### 3.2.1. Monocyclic Systems

**Scheme 15:**
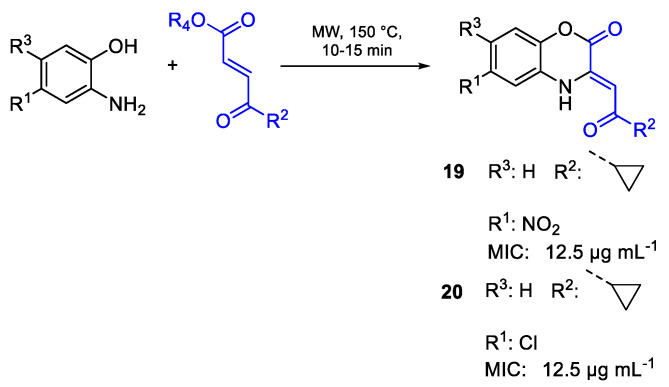
Synthesis of active molecules **19** and **20**.

Benzo[1,4]oxazines and 2-oxo-benzo[1,4]oxazines have presented different biological activities such as antibacterial, anti-inflammatory, antihypertensive, and antifungal [54]. Sharma et al. (2017) synthesized 2-oxo-benzo[1,4]oxazines incorporating different pharmacologically essential cyclic substituents. (*Z*)-3-(2-oxo-2-substituted-ethylidene)-3,4-dihydro-2*H*-benzo[*b*][1,4]oxazin-2-one type compounds were obtained using microwave irradiation under a cyclo condensation reaction between 2-aminophenols and 1,2-unsaturated ketones with yields from 80 to 95% (Scheme 15). The evaluation of antifungal activity against *FOX* showed that compounds **19** and **20** presented MIC values of 12.5 µg/mL than ketoconazole (Table 1) used as a positive control. SAR studies show that scaffolds of cyclopropyl and cyclohexyl phenyl substructures containing nitro groups in 2-oxo-benzo[1,4]oxazines increase antifungal activity [55].

**Scheme 16:**
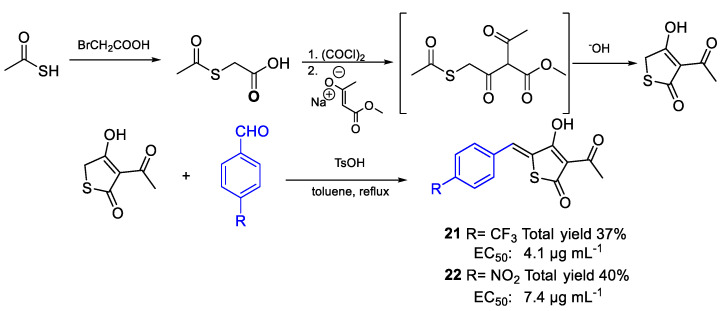
Synthesis of active molecules **21** and **22**.

Lv et al. (2018) reported a synthetic protocol to access 3-acyltetronic acid analogs, using thioacetic acid as the starting material. This protocol employed a linear strategy, in which was thioacetic acid reacted against bromoacetic acid in the first step. The obtained product was treated with oxalyl chloride followed by a Claisen condensation with the sodium salt of methyl acetoacetate to produce the respective γ-mercaptodiketoester. The latter suffered cyclization to provide the 3-acetyl-4-hydroxy-5*H*-furan-2-ones. Finally, the desired compounds were prepared by a condensation reaction between 3-acetyl-4-hydroxy-5*H*-furan-2-ones and aromatic aldehydes, obtaining 60 to 80% yields (Scheme 16). The evaluation of the antifungal activity against *FOX f.* sp. *lycopersy* of the synthesized compounds was determined at a concentration of 50 µg/mL according to the mycelial growth rate method. Based on these results, there was determined the mean effective concentration for each compound (EC_50_). Compounds **21** and **22** showed promising activity at *in vitro* level. Compound **21** presented an EC_50_ value of 4.1 µg/mL comparable with the positive control (azoxystrobin, EC_50_ 4.3 µg/mL). Analysis of the structure–activity relationship indicated that the presence of a trifluoromethyl group leads to better antifungal activity [56].

**Scheme 17:**
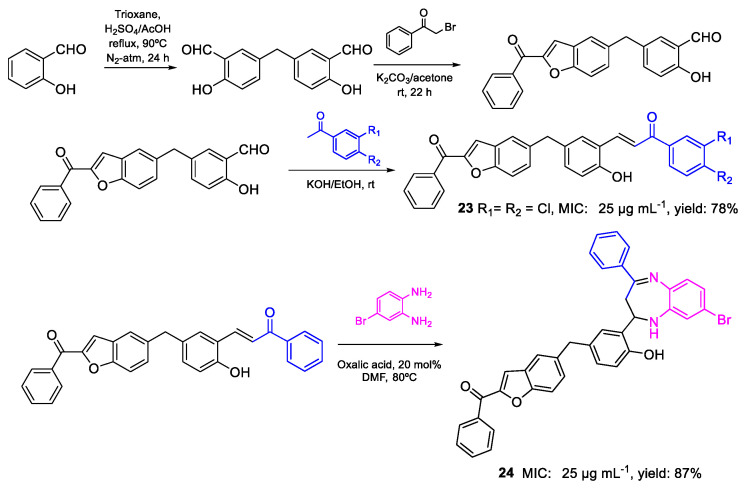
Synthesis of active molecules **23** and **24**.

The 1,4-benzodiazepine analogs have been widely studied due to their interesting pharmacological activities, low toxicity, and uses as a precursor developing powerful bioactive agents such as antibacterial, antidepressant, analgesic, anti-inflammatory, antifungal agents [57,58,59]. Shankar et al. (2016) synthesized a new series of {5- [4-hydroxy-3-(4-phenyl-2,3-dihydro-1*H*-benzo[*b*][1,4]diazepin-2-il)benzyl]-benzofuran-2-yl}(phenyl) methanone by condensation of (*E*)-3-{5-[(2-benzoylbenzofuran-5-yl)methyl]-2-hydroxyphenyl}-1-phenylprop-2-en-1-one with several substituted 1,2-phenylenediamines in the presence of oxalic acid as a catalyst with yields of 60 to 85% (Scheme 17). The evaluation of the antifungal activity against *FOX* showed that compounds **23** and **24** presented inhibition halos of 28 and 32 mm, respectively, values that were better than the positive control Nystatin (23 mm) [60].

**Scheme 18:**
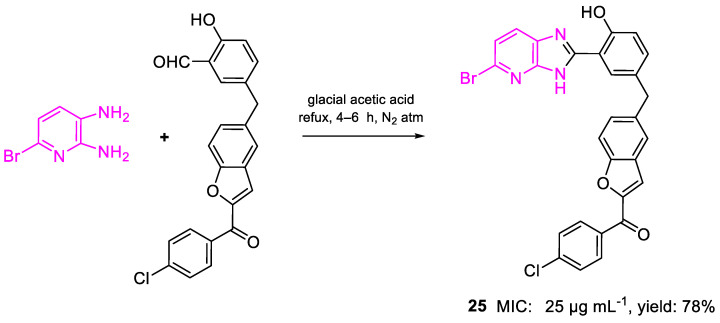
Synthesis of active molecule **25**.

Continuing their research, Shankar et al. (2018) carried out the condensation of 5-((2-benzofuran-5-yl)methyl)-2-hydroxybenzaldehyde with various substituted 1,2-diaminopyridines in the presence of glacial acetic acid under conventional reflux conditions, obtaining good to excellent yields (Scheme 18). In this case, the desired products were obtained through a 5-*exo*-*tet* cyclo condensation of a Schiff base intermediate. The compounds were evaluated at a concentration of 900 µg/mL against *F. oxysporum* by the disk diffusion method and by determining the MIC. Compounds **25** showed significant inhibitory activity with a MIC of **25** to 50 µg/mL [61].

**Scheme 19:**
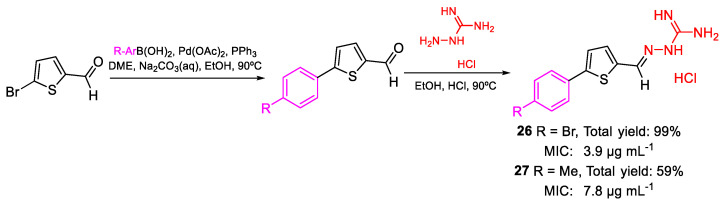
Synthesis of active molecules **26** and **27**.

Ajdačić et al. (2016) synthesized novel guanylhydrazones (iminoguanidines) with a thiophene ring as the core. The synthetic protocol employed a linear strategy, in which the first step involved the Suzuki cross-coupling reaction between *5*-bromo-2-thiophen*e*carbaldehyde and arylboronic acids using a palladium catalyst. The second step used a condensation reaction between aminoguanidine hydrochlorides and the *5*-aryl-2-thiophenecarbaldehyde affording the desired guanylhydrazone derivatives in good to excellent yield*s* (80–90%) (Scheme 19). The evaluation of the antifungal activity of the guanylhydrazone products was carried out against *FOX* AB18, showing that compounds **26** and **27**, which present methyl and bromine substituents in the fourth position of the phenyl ring, were the most active (Table 1). Presumably, the antifungal activity of these compounds would not depend on the electronic effect of the aromatic ring substituents but the hydrophobic effect [62].

**Scheme 20:**
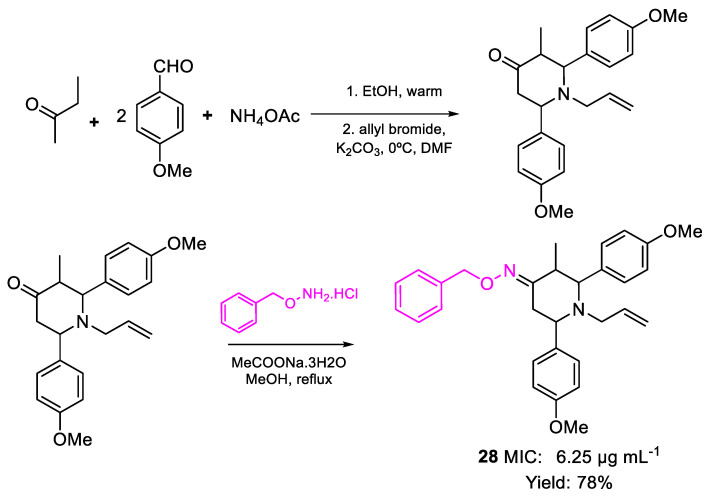
Synthesis of active molecule **28**.

Among a wide variety of heterocycles studied for medicinal purposes and the development of new molecules with pharmacological activity, piperidine derivatives possess a broad spectrum of biological activities, becoming an exciting heterocycles group [63]. Several studies carried out on 2,6-diarylpiperidine derivatives have demonstrated good antibacterial and antifungal activity. Narayanan et al. (2012) synthesized a series of 1-allyl-2,6-diphenylpiperidin-4-one oximes and 1-allyl-2,6-diphenylpiperidin-4-one *O*-benzyloximes by direct condensation of 1-allyl-2,6-diphenylpiperidin-4-ones and *O*-arylhydroxylamine hydrochloride in the presence of sodium acetate trihydrate, with yields from 70 to 85% (Scheme 20). The synthesized compounds were evaluated for antifungal activity against *FOX*. It was observed that compounds **28**, which present a substitution of *O*-benzyloxime in C-4, significantly inhibited this fungus, showing MIC values of 6.25 µg/mL, respectively [64].

#### 3.2.2. Polyheterocyclic Systems

**Scheme 21:**
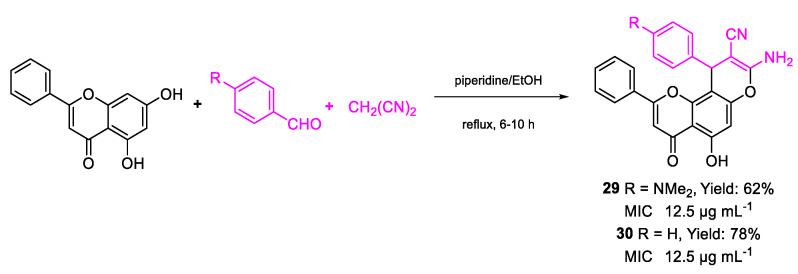
Synthesis of active molecules **29** and **30**.

Flavonoids and their derivatives are the most common families of vegetal secondary metabolites. They have received scientific interest due to their potential use as an antidiabetic, anticancer, antibacterial, antiviral, anti-inflammatory, antiallergic agent, and vasodilator. Chromene is the structural scaffold that occurs in most of the compounds of the flavonoid family, where the pyran nucleus is predominant in this type of natural product. Ramesh et al. (2015) have paid attention to chrysin, a natural flavone reported exhibiting numerous biological activities, including anticancer activities, anti-inflammatory, antioxidant, and antiallergic. For this reason, they focused on the synthesis of pyrano[2,3*H*]chrysin-type compounds through the multicomponent reaction between chrysin, aromatic aldehydes, and malononitrile [65]. The synthetic strategy started from chrysin, obtained using the procedure reported in the literature [66,67,68,69]. Then, the chrysin was treated with aromatic aldehydes and malononitrile in the presence of a catalytic amount of piperidine in ethanol, obtaining the desired products (Scheme 21). Aryl aldehydes with electron-withdrawing groups reacted rapidly with higher yields than the aryl aldehydes with electron-donor groups. The synthesized compounds were evaluated for their antimicrobial activity in vitro against a panel of bacterial and fungal strains, including *FOX*, showing promising biological activities for compounds **29** and **30**. The results indicated that the compounds containing phenyl, 4-methyl, 3-fluoro, 4-methyl, and 4,4-dimethylaminophenyl groups, respectively, in position 10, showed potent antimicrobial activity, which is further supported by their coupling studies molecular.

**Scheme 22:**
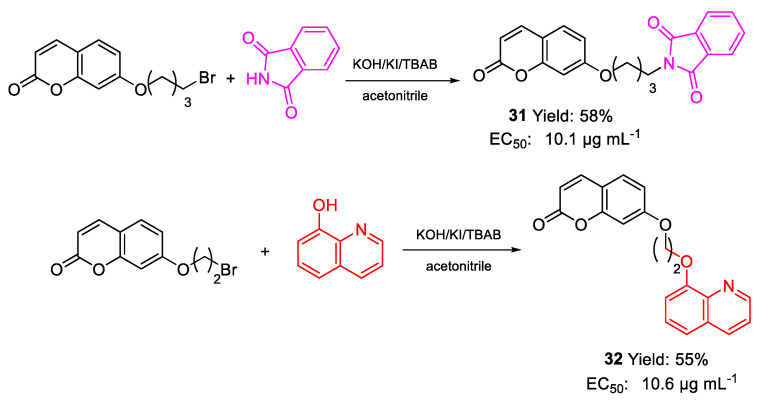
Synthesis of active molecules **31** and **32**.

Pan et al. (2017) obtained derivatives of umbelliferone, a known naturally occurring skeleton with a broad spectrum of bioactivity [70]. Umbelliferone has been identified as a vital allelochemical of Stellera chamaejasme. Furthermore, the antifungal properties of umbelliferone derivatives have been widely studied. Some tactics were provided to develop phytosanitary agents by modifying the target at the C-7 hydroxyl group of umbelliferone [71]. A series of C-7 *O*-substituted hydroxycoumarins and umbelliferone derivatives were obtained from bromoalkoxycoumarins through a one-pot alkylation reaction of phthalimide in the presence of KOH, KI, and TBAB in acetonitrile under reflux conditions. To obtain compound **32**, a mixture of quinoline derivatives and the respective halogenated umbelliferone derivatives reacted under the same conditions (Scheme 22). The biological activity against *Alternaria alternata*, *Alternaria solani*, *Botrytis cinerea*, and *FOX* showed that the synthesized derivatives, compared to umbelliferone, exhibited a better inhibitory effect. Compounds **31** and **32** showed the lowest EC_50_ values against *F. oxysporum* (Table 1). The results allowed to conclude that the modification in the C-7 hydroxy of umbelliferone could be a promising way to improve antifungal. The study of structure–activity relationships suggested that a C-4 methyl in umbelliferone contributed to fungal activity. The terminal residues and the most extended chain length were strongly responsible for the antifungal activities.

**Scheme 23:**
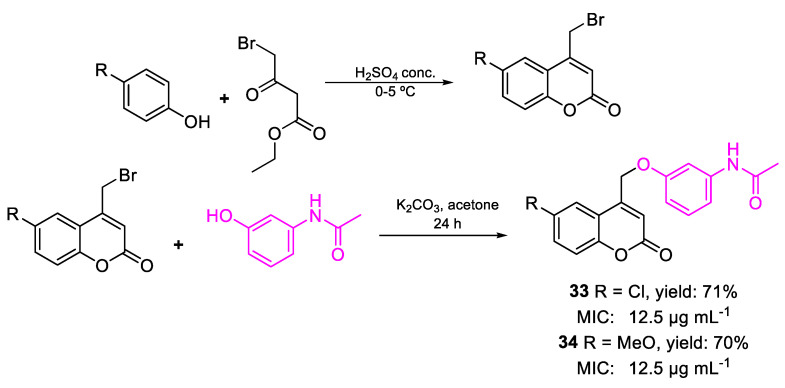
Synthesis of active molecules **33** and **34**.

Coumarins are an essential class of oxygenated heterocycles, which have received significant interest given their antibacterial, anti-inflammatory, antioxidant, anthelmintic, anticancer, and anti-HIV activities [72]. Especially, 4-substituted coumarin derivatives have exhibited antiplatelet, antituberculous, antioxidant, and cytotoxic activities. For some coumarin ethers in position 7, their behavior has been shown as potent monoamine oxidase inhibitors (MAO). Makandar et al. (2012) have observed that the binding of biocompatible fragments such as vanillin and paracetamol through an ether bond at the C-4 position of coumarin leads to molecular motifs with potential dual fluorescence and anti-inflammatory properties [73]. Thus, 4-aryloxymethylcoumarins derived from some substituted phenols were obtained by Pechmann cyclization with ethyl 4-bromoacetoacetate using sulfuric acid as a condensing agent [74]. The ethers were obtained under standard conditions from mixtures of resorcinol and anhydrous potassium carbonate in dry acetone, to which 6-methyl-4-bromomethylcoumarin was subsequently added (Scheme 23). The antifungal activities of the synthesized compounds were evaluated against the following standard fungal strains: *Candida albicans*, *Aspergillus fumigatus*, *Aspergillus niger*, *Penicillium chrysogenum*, *Mucor fuscus*, and *FOX* in dimethyl sulfoxide (DMSO) by the serial dilution method. The results revealed that compounds **33** and **34**, containing chlorine and methoxy substituents at the C-6 position of coumarin, showed higher activity than the others (Table 1). The activity of the chloro-substituted compounds is comparable to that of fluconazole in some cases.

**Scheme 24:**
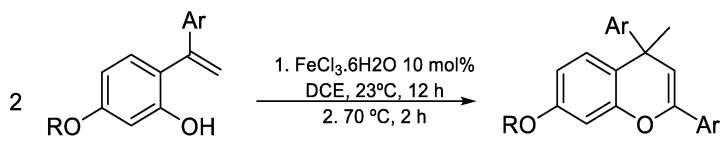
Synthesis of poly-substituted 4*H*-chromenes

Ma and Zhao (2018) reported the synthesis of poly-substituted 4*H*-chromenes via FeCl_3_ 6H_2_O catalyzed [4+2] cycloaddition followed by elimination of 1,1-diarylalkenes (Scheme 24) [75], in which *o*-quinone methide (*o*-QM) were used as interesting intermediates. *o*-QM can be generated from 1,1-diarylalkenes with an *ortho*-hydroxyl directing group promoted by Brønsted acids, which subsequently undergoes attack by various nucleophiles. This transformation involves a [4+2] cycloaddition followed by the elimination reaction of *meta*-methoxy-phenol to deliver a 4*H*-chromene bearing all-carbon quaternary center. Control experiments indicated that the palladium catalyst was not necessary for the transformation.

**Scheme 25:**
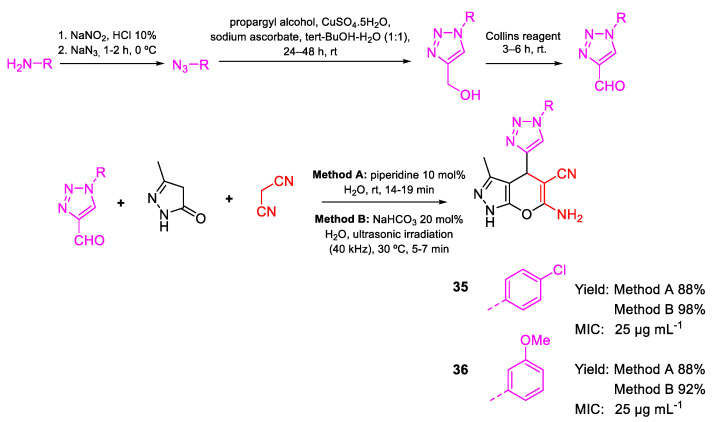
Synthesis of active molecules **35** and **36**.

Recently, Khare et al. (2019), based on the pharmacological activities of both pyrazole and pyran, carried out the synthesis and evaluation of 1,2,3-triazolyl pyrano[2,3-*c*]pyrazoles [76]. The three biologically active scaffolds, 1,2,3-triazole, pyrazole, and 4*H*-pyran, were joined into a single molecular framework using a three-component, one-pot synthesis strategy, resulting in novel 1,2,3-triazolyl pyrano[2,3-c]pyrazoles (Scheme 25). Initially, triazolyl aldehydes were prepared from anilines by a click chemistry approach. The reaction of triazolyl aldehyde, malononitrile, and pyrazolone using piperidine as catalyst afforded 1,2,3-triazolyl pyrano [2,3-c]pyrazole in a yield of 84%. A second methodology used sodium bicarbonate as the catalyst, and ultrasound irradiation allowed to obtain the desired compounds with yields between 80–92%, becoming a more ecological synthetic protocol. Most of the 1,2,3-triazolyl pyrano[2,3-c]pyrazole derivatives were evaluated against most fungal strains. Compounds **35** and **36** showed equivalent activity compared to the standard drug against *FOX*. In addition, it demonstrated that the antifungal activity varies with the substituent present in an aromatic unit of 1,2,3-triazolyl pyrano[2,3-c]pyrazole. Compounds substituted with 4-OMe, 3-OMe, 2-OMe, 4-Cl, and 3-NO_2_ showed excellent antifungal activity than compounds substituted with H, 4-Me, 3-Cl, 4-NO_2_, and 2-NO_2_.

**Scheme 26:**
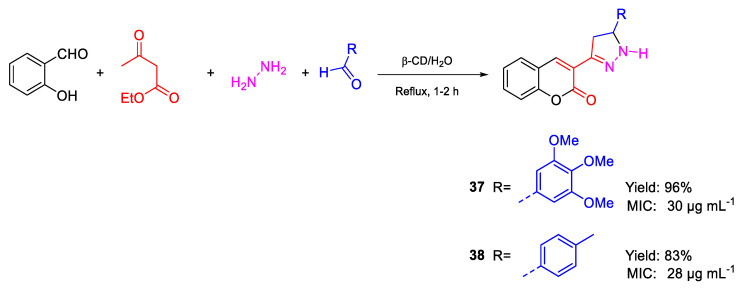
Synthesis of active molecules **37** and **38**.

Several researchers in the literature have predicted that “the union of the coumarin and pyrazoline pharmacophores would generate new molecular templates that would likely exhibit interesting biological properties” [77]. In particular, coumarin-linked pyrazoline pharmacophores have been reported to possess antitumor, antimalarial, and anticancer properties. Chate et al. (2019) synthesized new pyrazoline analogs integrated with coumarin (Scheme 26) and evaluated their possible behavior as antibacterial agents against D-alanine-D-alanine ligase (Ddl) in bacteria [78]. The one-pot reaction between salicylaldehyde, ethyl acetoacetate, hydrazine hydrate, and benzaldehyde in water was carried out using cyclodextrins (a-CD, b-CD, and g-CD) as a catalyst. Excellent results were obtained with b-CD as a catalyst even at 5 mol%. The results changed using 15 mol% of cyclodextrin, being observed higher yields of the products. The authors explained these results from host-host complexes, in which the reactants lodge within their lipophilic cavity using non-covalent interactions, providing indirect proof that b-CD behaves like a reactor. The antifungal activity in vitro of the synthesized derivatives against different pathogenic yeast and filamentous fungi was evaluated, determining the minimum inhibitory concentration (MIC) values. The results indicated that all the synthesized compounds showed good to moderate antifungal activity against the fungal strains tested. Compounds **37** and **38** showed MIC values of 28–30 mg/mL for *FOX*, showing similar activity to the standard drug miconazole.

**Scheme 27:**
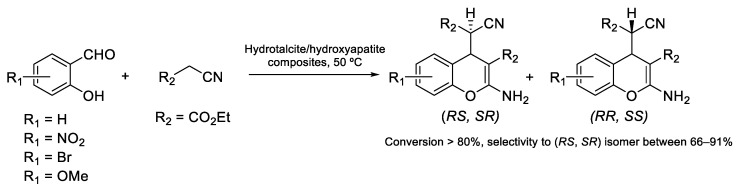
Synthesis of chromenes catalyzed by hydrotalcite/hydroxyapatite composites.

Recently, Velázquez-Herrera et al. (2020) employed hydrotalcite/hydroxyapatite composites as catalysts for the synthesis of chromenes, showing higher conversions (up to 78% after 2 h), under solvent-free and mild conditions [79]. The authors suggested that both basicity and porosity are important factors controlling the catalytic behavior. Basic sites in composites combined with high mesoporosity and SBET can promote the reaction between ethyl cyanoacetate and salicylaldehyde (Scheme 27). The reactivity of the latter depends on the electronic nature of the substituents.

**Scheme 28:**
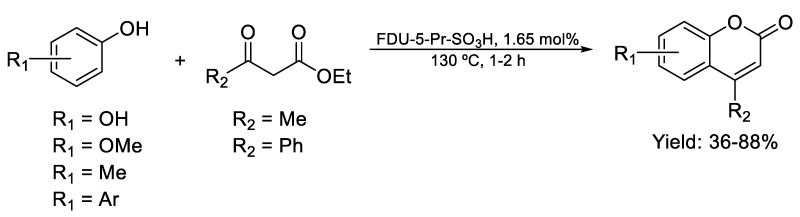
Synthesis of coumarin derivatives using FDU-5-Pr-SO_3_H material as catalyst.

Gonzalez-Carrillo et al. (2020) used FDU-5-Pr-SO_3_H material as the catalyst for the synthesis of coumarin derivatives via Pechmann condensation between β-keto-esters and substituted phenols (Scheme 28) [80]. The results indicate that using the proposed material as a catalyst reaches a high turnover frequency in the synthesis of coumarin derivatives via condensation of phenols and β-keto-esters with good to excellent yields using a wide variety of phenolic substrates. Nevertheless, it is essential to develop regeneration methods for this organic catalyst supported in mesoporous solid, allowing for excellent reaction conditions.

**Scheme 29:**
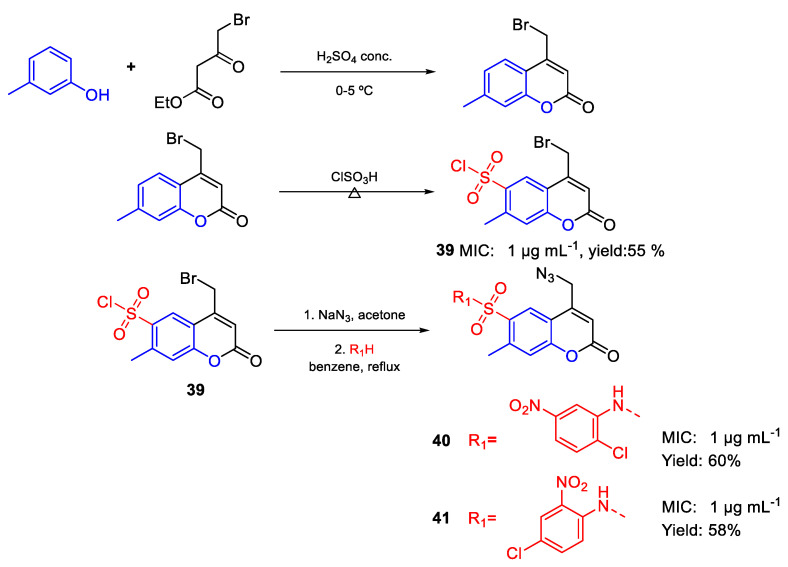
Synthesis of active molecules **39**–**41**.

Basanagouda et al. (2010) synthesized a series of new coumarins 6-sulfonamides using a linear strategy. The bromination of ethyl acetoacetate and the subsequent reaction against *m*-cresol under Pechmann cyclization conditions afforded a 4-bromomethylcoumarin, heated under reflux with an excess of chlorosulfonic acid to give 4-bromomethyl-7-methyl-coumarin-6-sulfonyl chloride. The latter was treated with sodium azide affording 4-azidomethyl-7-methylcoumarin-6-sulfonyl chloride. Finally, the sulfonamides were obtained by reaction against various aromatic amines in benzene under reflux conditions (Scheme 29). All sulfonamides were analyzed for their antifungal activity on *FOX* by the broth microdilution method. The compounds **39**–**41** showed better activity than the positive control (fluconazole MIC 8 μg/mL) with MIC values of 1 μg/mL [81].

**Scheme 30:**
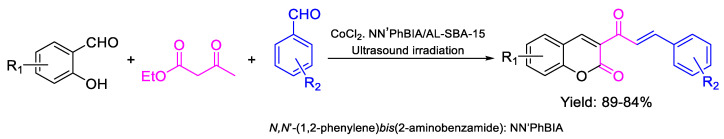
Synthesis of 3-cinamoyl coumarins.

Akbarzadeh and Safaei-Ghomi (2020) reported the synthesis of a mesoporous Al-SBA-15 modified support which included a *N*,*N’*-(1,2-phenylene)*bis*(2-aminobenzamide)dichloro cobalt complex. Its catalyst capacity was evaluated to synthesize 3-cinnamoyl coumarins via a three-component reaction between benzaldehydes, salicylaldehydes, and ethyl acetoacetate (Scheme 30) [82]. Ultrasound irradiation combined with this material as a catalyst allowed to obtain the desired compounds in excellent yields. This novel catalyst has gained attention by applying different functional groups on the salicylaldehyde and aromatic aldehydes.

**Scheme 31:**
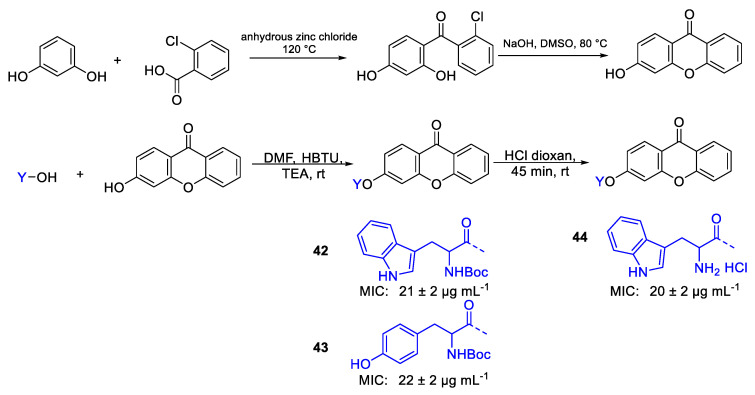
Synthesis of active molecules **42**–**44**.

Xanthones are a class of heterocyclic compounds widely distributed in nature. Much research has focused on isolation from natural products or the synthesis of new drugs [83]. Chen et al. (2017) synthesized a new series of xanthones conjugated with amino acids. These have recently been used due to their pharmaceutical applications, good bioavailability, permeability, low toxicity, and pharmacokinetics. The synthesis strategy used involves a first step in which resorcinol reacts with 2-chlorobenzoic acid in the presence of zinc chloride as a catalyst to obtain benzophenone derivatives with hydroxyl groups in positions 3 and 4. The use of sodium hydroxide and heating allowed cyclization of benzophenone derivatives to a xanthone tricyclic system. Finally, the derivatization of the remaining hydroxyl group by reaction with derivatives of *L*-tryptophan and *L*-tyrosine in the presence of HBTU (2-(1*H*-benzotriazol-1-yl)-1,1,3,3-tetramethyluronium hexafluorophosphate: Hexafluorophosphate Benzotriazole Tetramethyl Uronium) allowed expanding the group of xanthones being obtained heterofunctionalized polycyclic systems. These new derivatives were obtained with good yields (Scheme 31) and were evaluated against *FOX*. The most active compounds against *FOX* were **42**–**44**. The xanthone precursors with amino acid moieties such as phenylalanine, tyrosine, tryptophan, cysteine, and proline, showed excellent antifungal activity compared to glycine, alanine, valine, leucine, and isoleucine. The presence of aromatic amino acids explained these results due they play an important role in anchoring the amino acids/peptides to cell membranes, having high aromaticity, hydrophobicity, light stability, and an amphiphilic structure [84].

**Scheme 32:**
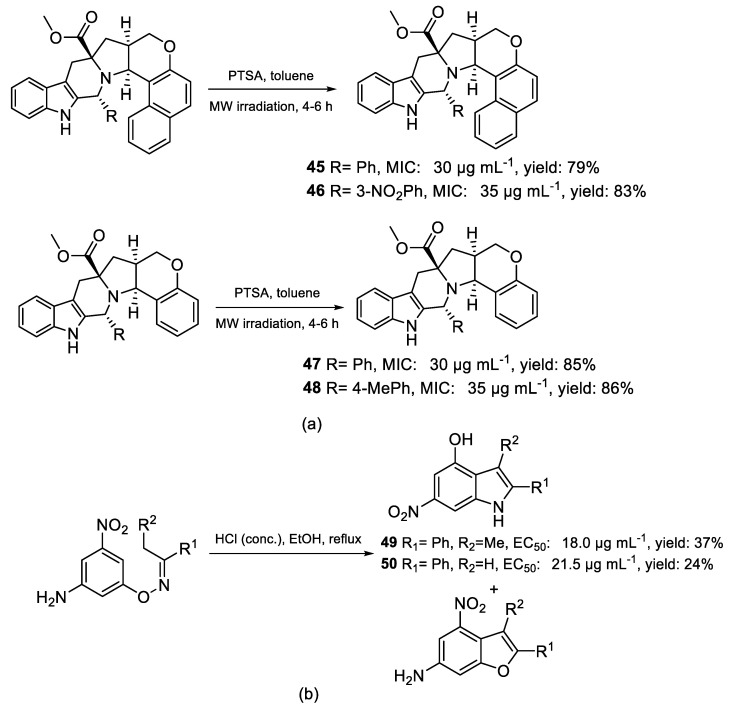
Synthesis of active molecules: (**a**) obtention of **45**–**48** Pictet-Spengler cyclization under microwave radiation; (**b**) obtention of **49** and **50** from *O*-aryl oximes.

The indole ring is found in abundance in naturally occurring compounds and constitutes one of the best-known heterocycles [85,86]. A widespread range of bioactivities has been attributed to this privileged scaffold, the protagonist of multiple alkaloids, drugs, and agrochemicals. Arumugam et al. (2012) focused on constructing indolizino-indole heterocycles, a fused system of interest to the pharmaceutical industry [87]. They employed an intramolecular 1,3-dipolar cycloaddition to assemble the chromeno-pyrrole fused system, followed by forming the indolizino-indole moiety via a Pictet-Spengler cyclization under microwave radiation. The MIC values against FOX of this novel series of fused heterocycles ranged from 59.93 to 236.78 µM. Compounds **45**–**48** exhibited the best inhibitory activity (Table 1), even higher than control drug Carbendazim (MIC = 104.61 µM). Another example constitutes a couple of 2-arylindoles (**49** and **50**, Table 1) that presented fungicidal activity towards several taxonomic classes of fungi, including *FOX* [88]. Kokurkina et al. (2011) aroused interest in this type of substituted indoles, mainly because they constituted an excellent synthetic scenario for applying for their previous research work on transformations of explosive aromatic compounds. Substituted 2-arylindoles are known to be bioactive molecules with antibiotic properties. In this sense, to obtain access to a series of these indolic analogs, trinitrobenzene and 1,3-dinitro-5-trifluoromethylbenzene were employed as starting material and successfully converted in the different ketoximes. Then, after a controlled reduction, they used an acid-mediated regioselective intramolecular [3,3]-sigmatropic rearrangement with a concomitant cyclization as the critical step. However, it is worth noting that the cyclization step was not chemoselective, resulting in a mixture of arylbenzofurans and arylindoles (Scheme 32). Interestingly, the former did not show any remarkable fungicidal activity, while some arylindoles had higher values than commercial fungicide triadimefon.

**Scheme 33:**
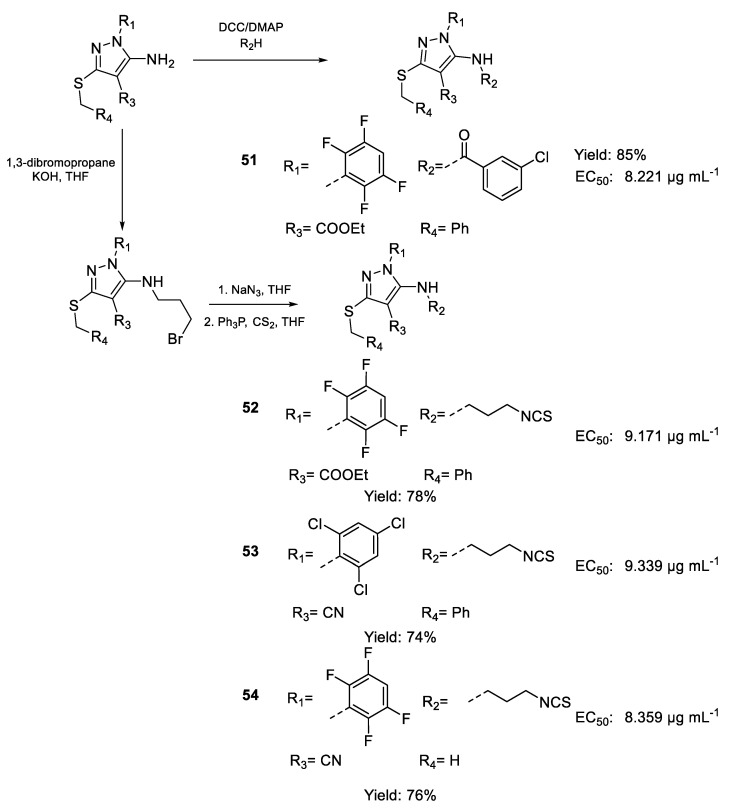
Synthesis of active molecules **51**–**54**.

In recent years, substituted pyrazole derivatives have gained considerable attention due to broad biological activity [89,90]. The pyrazole ring is a widely known pharmacophore used for fungicide design. Liu et al. (2014) designed and synthesized 32 new pyrazole derivatives with yield percentages more significant than 90% (Scheme 33). The synthesis of compound **51** was carried out from the amidation of the amino group of the precursor type 3-(arylmethyllthio)-1*H*-pyrazole by reaction with the respective chlorobenzoic acid in the presence of DMAP (4-dimethylaminopyridine). Compounds **52**–**54** were obtained using a different method, in which initially the amino group of the precursor type 3-(arylmethylthio)-1*H*-pyrazole was alkylated with 1,3-dibromopropane and subsequently, by reaction with sodium azide and then with triphenylphosphine and carbon disulfide, carried out the formation of a new isothiocyanate group in the side chain. In vitro tests of antifungal activity against *F. oxysporum* showed an effect greater than 80% and EC_50_ values of 6 to 9 μg/mL. Most active compounds were established as **52**–**54**. The results obtained from the antifungal activity of the pyrazole derivatives showed that the compounds with the presence of the cyano group and an amide group appear to improve the biological activity [91].

**Scheme 34:**
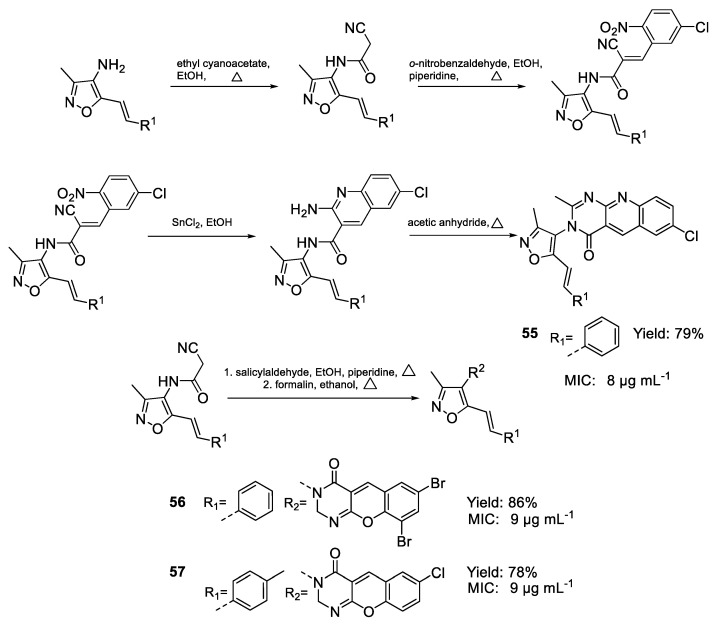
Synthesis of active molecules **55**–**57**.

Rajanarendar et al. (2012) synthesized some new pyrimidoquinolines and chromenoquinolines linked to isoxazole from isoxazolyl cyanoacetamides were studied as useful precursors to build different heterocyclic systems [92]. Furthermore, the active hydrogen at the C-2 position of these compounds can participate in condensation and substitution reactions. The synthesis protocol of compound **55** employs a linear strategy. In the first step, isoxazole-3-amine reacted with ethyl cyanoacetate towards an amide-type derivative, which was subsequently treated with *o*-nitrobenzaldehyde in piperidine, carrying out a condensation reaction and giving rise to the formation of a 1,2-unsaturated nitrile. Subsequently, the latter underwent intramolecular cyclization in SnCl_2_, forming a fused pyridine system after the previous reduction of the nitro group, to finally underwent a new cyclization in the presence of acetic anhydride, leading to the formation of a pyrimidine-4-one nucleus. Compounds **56** and **57** were obtained using a similar method; however, different salicylaldehydes were used instead of *o*-nitrobenzaldehydes to obtain the pyran nucleus instead of pyridine (Scheme 34). These derivatives were obtained as good yields (70–85%) and were evaluated against *F. oxysporum*, showing that compounds **55**–**57** are significantly toxic for this fungus, showing values lower than the positive control (clotrimazole MIC 28 µg/mg).

**Scheme 35:**
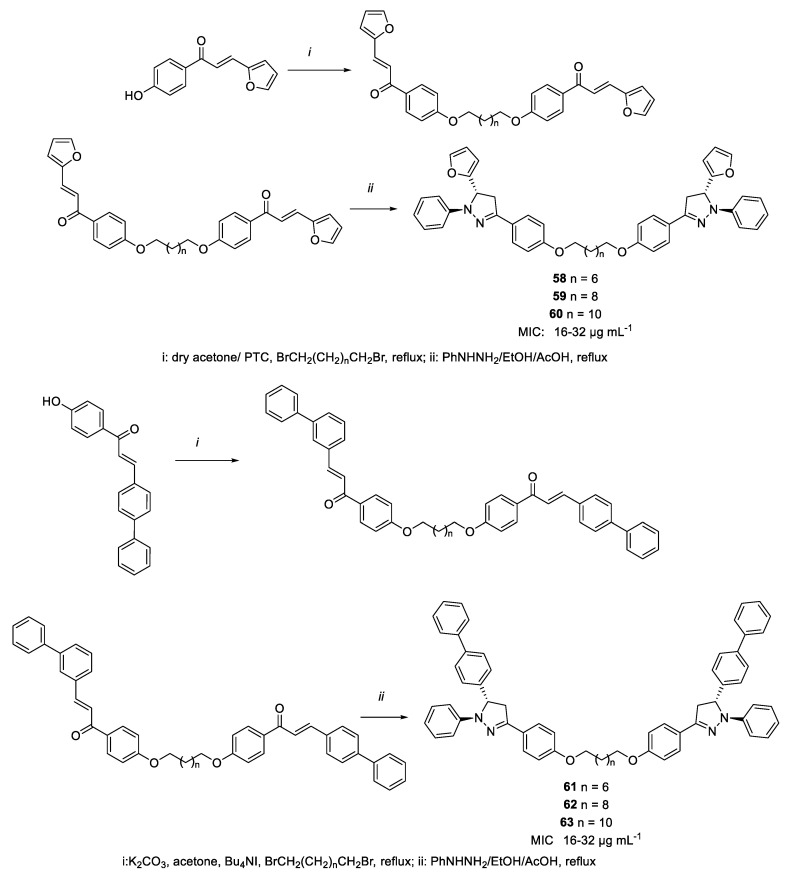
Synthesis of active molecules **58**–**63**.

Five-membered heterocycles compounds containing two nitrogen atoms, such as substituted pyrazolines (2-pyrazolines), have played an essential role in discovering new compounds that exhibit different biological activities present in some pharmacologically active molecules such as antitumor, antioxidant, antiviral, and antimicrobial. Yusuf and Solanki (2017) synthesized new *bis*chalcones prepared from aliphatic chains of different lengths and their transformations into *bis*pyrazolines with yields between 50 to 70% (Scheme 35). These derivatives were evaluated to determine their antifungal activity against *FOX* by determining the minimum inhibitory concentration (MIC). The results showed compounds **58**–**60** have moderate antifungal activity with MIC values of 16 to 32 µg/mL [93]. In another study, Yusuf and Solanki (2019) reported synthesizing a series of new *bis*pyrazolines from the cyclization reactions of *bis*chalcones with phenylhydrazine by refluxing under alcoholic alkaline conditions (KOH/EtOH). These *bis*heterocycles were obtained with yields between 70 to 80%. In vitro antifungal evaluation against *F. oxysporum* showed that these compounds have moderate activity, *bis*pyrazoline **62**, which exhibited the best activity (MIC between 16 µg/mL) and their corresponding intermediates *bis*chalcones [94].

**Scheme 36:**
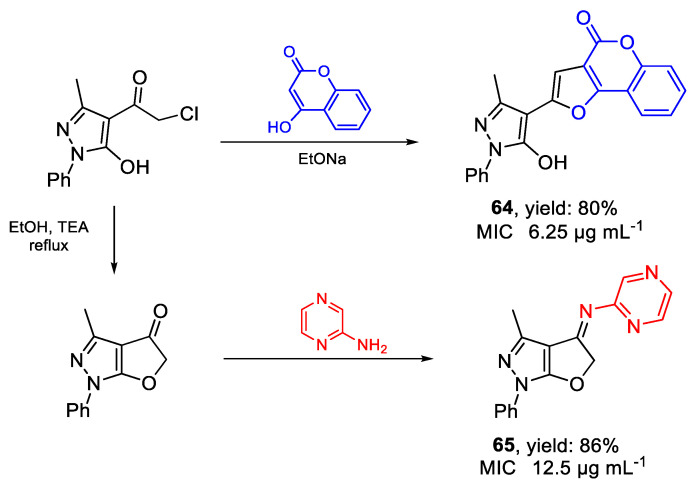
Synthesis of active molecules **64** and **65**.

Bondock et al. (2011) proposed to study the structural variation through the union of some biologically active heterocycles (quinoxaline, benzothiazine, benzoxazine, furan, benzofuran, and furan[3,2-*c*]coumarin) in position 4 of the central pyrazole nucleus, as well as the construction of some furo[2,3-*c*]pyrazoles. They obtained a series of new functionalized 4-hetarylpyrazoles and furo[2,3-*c*]pyrazoles with 70 to 80% yield percentages (Scheme 36). The antifungal activity tests were carried out on *FOX* (ATCC16417) showed that compounds **64**–**65** presented MIC values between 6.25–12.5 µg/mL, due to the incorporation of the furan or benzofuran group in position 4 of the pyrazole and by the low electron density in ring systems [95].

**Scheme 37:**
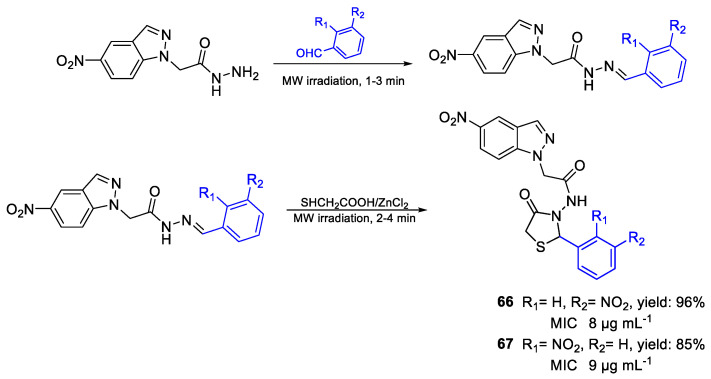
Synthesis of active molecules **66** and **67**.

Microwave-assisted synthesis of heterocyclic compounds is an efficient strategy that has become a powerful tool for green chemistry. The reaction time can be reduced, and the yields of the products usually tend to be improved. Upadhyay et al. (2010) synthesized *N*-[(4-oxo-2-substitutedaryl-1,3-thiazolidine)-acetamidyl]-5-nitroindazoles from 2-(5-nitro-1*H*-indazol-1-yl)acetohydrazide in a synthetic strategy with two steps: first, 2-(5-nitro-1*H*-indazol-1-yl)acetohydrazide reacted against aromatic aldehydes to afford Schiff bases, which reacted with mercaptoacetic acid in the presence of ZnCl_2_ under microwave irradiation (MW) to afford the desired products (Scheme 37). The reactions were carried out both by the conventional method and by microwaves. It was evident that the percentages of yields by the traditional method of reflux were lower (60–80%) than those obtained with microwaves (80–95%). The results obtained on its antifungal activity against *FOX* showed that compounds **66** and **67** presented the best MIC values of 9 and 8 µg/mL, respectively. Its activity against this fungus may be due to a nitro group in the *ortho* and *meta* positions of the aryl ring [96].

**Scheme 38:**
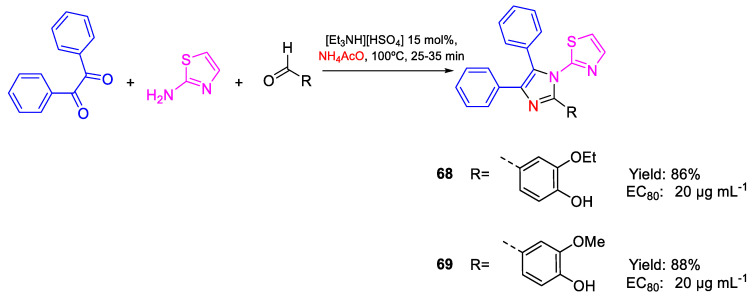
Synthesis of active molecules **68** and **69**.

Much research has focused on the search for compounds with antifungal potential that contain heterocycles in their structure. Within this large group are imidazole and thiazole, essential systems in medicinal chemistry due to their broad-spectrum, affinity against different targets, and a wide range of biological activities such as antitumor analgesics and antifungal agents [97,98]. Nikalje et al. (2017) synthesized a new series of imidazole-thiazole derivatives with good yields through a three-component condensation (Scheme 38). All the synthesized compounds were evaluated in vitro against *F. oxysporum* (NCIM1332). The results showed that compounds **68** and **69** had lower MIC values of 20 µg/mL than positive controls that presented MIC_80_ values of 40 and 50 µg/mL, respectively. The structure–activity relationship (SAR) studies showed that the 4,5-diphenyl imidazole and thiazole groups are responsible for the biological activity [99].

**Scheme 39:**
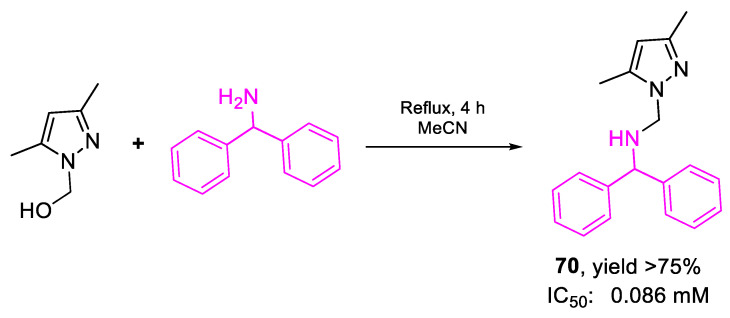
Synthesis of active molecule **70**.

Abrigach et al. (2017) prepared some pyrazole derivatives using several primary amines such as 1,1-diphenylmethylamine with good yields (Scheme 39). Five of these derivatives were selected to evaluate their antifungal activity against *F. oxysporum f.* sp. *albedinis*. Compound **70** showed excellent efficacy with an IC_50_ value of 0.086 mM, respectively, which can be explained due to the presence of two phenyl rings [100].

**Scheme 40:**
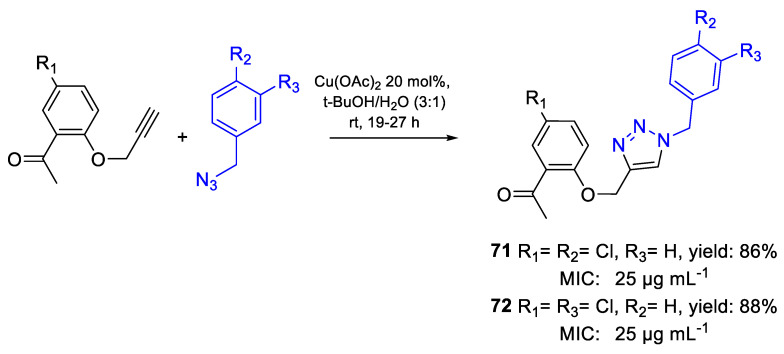
Synthesis of active molecules **71** and **72**.

Recently, click chemistry has emerged as a fast and robust method for synthesizing new biologically active compounds. Shaikh et al. (2016) synthesized novel compounds focused on acetophenones based on 1,4-disubstituted 1,2,3-triazoles through the fusion of benzyl azides and the alkyne group attached to the acetophenones using cupric acetate as a catalyst. The products were obtained in yields of 86 to 90% (Scheme 40). The compounds were evaluated against FOX. Compound **71** was the most active with a MIC of 12.5 µg/mL and equipotent to the drug miconazole (MIC 25 µg/mL) used as a positive control [101].

**Scheme 41:**
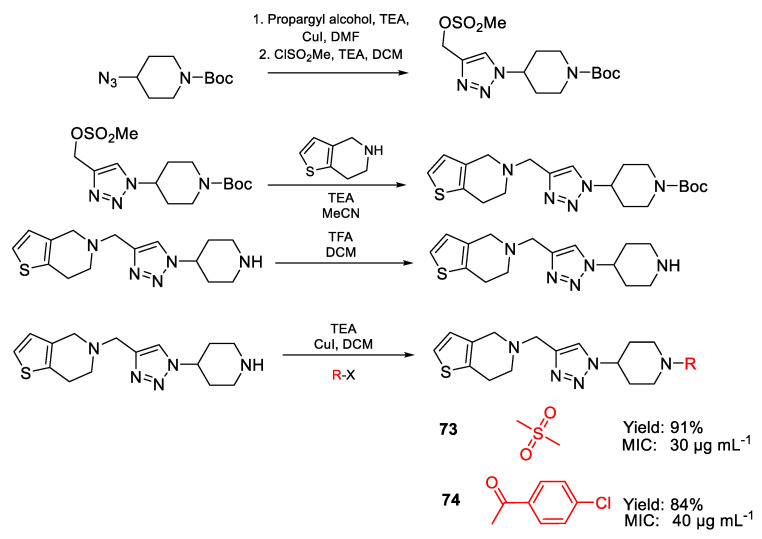
Synthesis of active molecules **73** and **74**.

In 2013, Darandale et al. reported for the first time the synthesis of heterocyclic molecules with fused rings of 1,2,3-triazoles, piperidines, and thienopyridine with yields of 82 to 91%. The synthesis protocol for these compounds starts from an azide derived from piperidine, which reacts with propargyl alcohol undergoes a [3+2] cyclization reaction, forming a 1,2,3-triazole nucleus. The presence of mesyl chloride leads to a methyl sulfonic group at the 4-position of the 1,2,3-triazole ring. This heterocyclic system functions as a precursor to compounds **73** and **74**, in such a way that it is initially reacted with 4,5,6,7-tetrahydrothieno[3,2-*c*]pyridine by nucleophilic substitution, then by reaction with trifluoroacetic acid, the carbamate group is removed. Finally, the free amino group reacts with an electrophilic generated starting from mesyl chloride or aryl acyl chloride, allowing the desired compounds’ formation (Scheme 41). All compounds were screened for their antifungal activity against *F. oxysporum* (NCIM1332). Compound **73** was the most active in this series, presenting a MIC value of 30 µg/mL. The structure–activity relationship (SAR) revealed fascinating data on the variation of the activity on this fungus. The presence of the sulfonyl group in compound **73** significantly improved the activity compared to other series compounds [102].

**Scheme 42:**
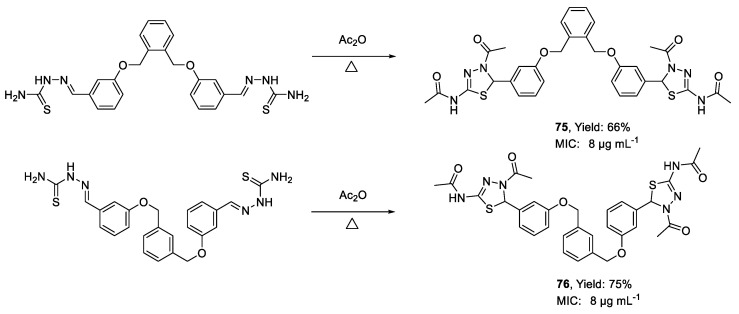
Synthesis of active molecules **75** and **76**.

1,3,4-thiadiazoline, 1,2,4-triazoles, and 1,3,4-oxadiazoles are heterocycles related to biological properties such as antifungal, anti-inflammatory, antimalarial, anti-HIV activity, anti-TB properties, antimicrobial, anticancer, antiviral, antineoplastic, CNS depressant, and tyrosinase inhibitory activities [103,104,105,106]. Yusuf, Kaur, and Jain (2014) carried out the cyclization of *bis*tiosemicarbazones in the presence of acetic anhydride under reflux conditions to obtain 1,3,4-thiadiazoline [107]. The *bis*tiosemicarbazones were obtained from the reactions of dibenzaldehydes with thiosemicarbazide at reflux in the presence of dry EtOH/HCl. The latter were prepared from the O-alkylation of 3-hydroxybenzaldehyde with dibrominated hydrocarbons under alkaline conditions with good yields. Finally, the heterocyclic system can be formed by intramolecular cyclo condensation assisted by acetic anhydride (Scheme 42). The antifungal activity of the synthesized compounds, both *bis*triosemicarbazones, and 1,3,4-thiadiazoline, were determined in vitro using the serial dilution method against five strains of fungi: *Aspergillus janus* (MTCC 2751), *Aspergillus niger* (MTCC 281), *FOX* (MTCC 2480), *Aspergillus sclerotiorum* (MTCC 1008), and *Penicillium glabrum* (MTCC 4951). Fluconazole was used as a reference drug for comparison and DMSO as a negative control. Compounds such as **75** and **76** were active against strains *B. subtilis*, *A. janus*, *P. glabrum*, *A. niger*, and FOX. In general, *bis*tiadiazolines were found to be more biologically active than their corresponding *bis*tiosemicarbazones. In general, *bis*tiadiazolines were found to be more biologically active than their corresponding *bis*tiosemicarbazones. Presumably, the internal spacer geometry significantly affected the antimicrobial behavior of *bis*thiadiazoline and the *bis*heterocyclics linked through the aromatic moiety (compounds **75** and **76**). It was concluded that derivatives with o-xylene, p-xylene, and biphenyl fragments exhibited better activity than compounds involving olefinic and alkyne chains.

**Scheme 43:**
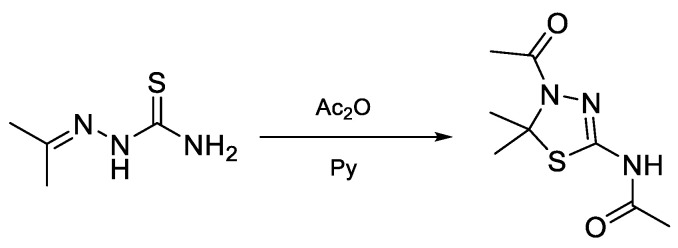
Synthesis of thiazolidine from isopropylidene thiosemicarbazide and acetic anhydride.

Recently, El-Atawy et al. (2019) reported the synthesis of alkylidene thiosemicarbazide without catalyst using water as solvent [108]. This report is exciting since the thiosemicarbazide-type compounds are supposed to be excellent precursors of heterocycles of the triazolidine-3-thione type. However, experiments carried out from semicarbazide and acetone gave thiosemicarbazide isopropylidene in a 95% yield. The subsequent reaction of this compound with different aldehydes in an aqueous medium allowed to give arylidene thiosemicarbazide instead of the expected arylidene isopropylidene thiosemicarbazide in good to excellent yields (72–95%). These results suggest that the successful synthesis reported for triazolidine-3-thione in various reports was not performed by cycloaddition of thiosemicarbazide or cyclo condensation of thiosemicarbazide with aldehyde or ketones, implying that the 1,2,4-triazolidin-3-thione scaffold is easily accessible by a three-component reaction between hydrazines, aldehydes or ketones and potassium thiocyanate in hydrochloric acid or a reaction between isopropylidene thiosemicarbazide and acetic anhydride in pyridine to afford thiadiazoline (Scheme 43).

**Scheme 44:**
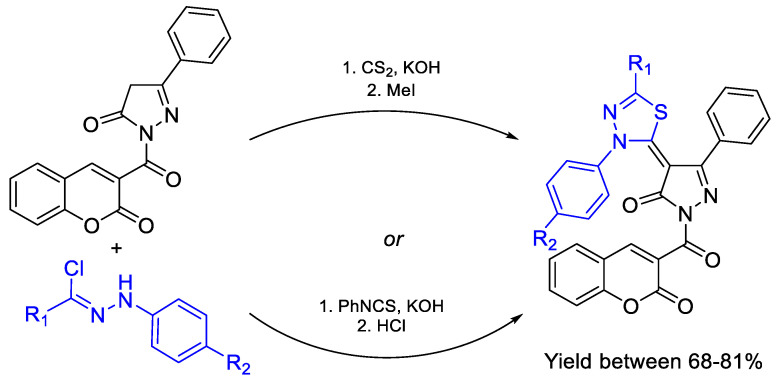
Obtention of 1,3,4-thiadiazole ring reported by Gomha.

Gomha et al. (2019) constructed five-membered heterocycles with multiple heteroatoms as nitrogen and sulfur from readily available starting materials and reagents [109]. The reaction of 1-(2-oxo-2*H*-chromene-3-carbonyl)-3-phenyl-1*H*-pyrazol-5(4*H*)-one with phenylisothiocyanate in alcoholic potassium hydroxide or carbon disulfide in basic medium afforded thioanilide and methylthio derivatives, respectively. Treatment of the latter compounds with various hydrazonoyl halides resulted in the construction of thiadiazole moiety linked to the pyrazole ring (Scheme 44).

**Scheme 45:**
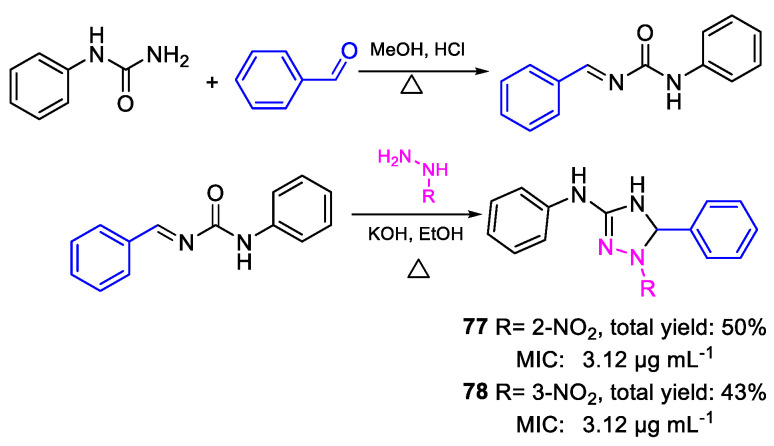
Synthesis of active molecules **77** and **78**.

Then, Yusuf and Thakur (2019) carried out the cyclization of several Schiff bases and phenylhydrazine by refluxing in an alkaline medium (KOH/EtOH mixture) with good yields affording novel 1,2,4-triazoles [110]. The precursor Schiff bases were prepared by condensation reactions of aromatic aldehydes with *N*-phenylurea using methanol as a solvent in an acid medium (Scheme 45). The obtained 1,2,4-triazoles showed excellent antifungal activities against various phytopathogens such as *FOX*. Biological activities were determined as minimum inhibitory concentrations (MICs) using the serial dilution technique and the lowest concentration required to stop the growth of bacterial and fungal strains. Amoxicillin and fluconazole were used as standard drugs. Compounds **77** and **78** showed significant activity (MIC of 6.25 μg/mL) against *K. pneumonia*, *P. aeruginosa*, *E. coli*, *F. oxysporum*, and *A. sclerotiorum*.

**Scheme 46:**
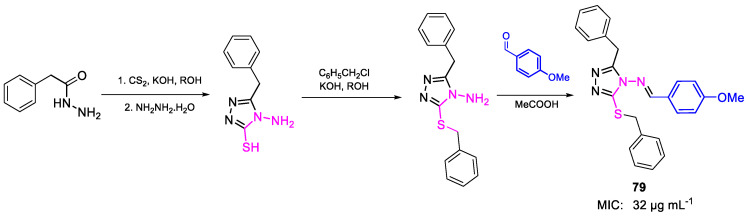
Synthesis of active molecule **79**.

*Combinatorial chemistry* is a technique by which many structurally different molecules can be synthesized in a short time and evaluated since it is a fast and interactive process. Bhatia et al. (2010) carried out the combinatorial synthesis of 1,2,4-triazole derivatives with good yield percentages, which were evaluated against *F. oxysporum* (NCIM-1008). Compound **79** presented the lowest MIC values (32 µg/mL) [111]. The established synthesis method uses 2-phenylacetohydrazide as a precursor, which reacts with carbon disulfide in a basic medium and subsequently with hydroxylamine, achieving the formation of the 1,3,4-triazole heterocyclic system. In a second stage, the thiol group in position 2 is derivatized towards thiobenzylether. Finally, by reaction of the amino group in position 1 with an aromatic aldehyde leads to the formation of the respective Schiff’s base (Scheme 46).

**Scheme 47:**
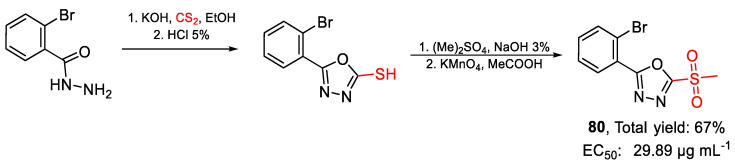
Synthesis of active molecule **80**.

Xu (2011) has synthesized several sulfones with 1,3,4-oxadiazole residues [112]. Previous *in vitro* bioassays revealed that the compounds 2-(methylsulfonyl)-5-(3,4,5-trimethoxyphenyl)-1,3,4-oxadiazole and 2-(benzylsulfinyl)-5-(3,4,5-trimethoxyphenyl)-1,3,4-oxadiazole have high antifungal activity against 10 types of fungi, with EC_50_ values ranging from 19.9 μg/mL to 93.3 μg/mL, being equivalent or more potent against fungi tested than the commercial agricultural fungicide hymexazol. The synthetic method uses an intermolecular cyclization between 2-phenylacetohydrazide and carbon disulfide in an acid medium, forming a heterocyclic nucleus of 1,3,4-oxadiazole. Finally, the thiol group is derivatized until the formation of the respective sulfone (Scheme 47). SAR studies suggested that 2-(methylsulfonyl)-1,3,4-oxadiazole is the central ring system that provides potent antifungal activities. So, it was postulated as a potential pharmacophore. The antifungal activity tests were performed at a 50 µg/mL concentration for all the compounds obtained, which exhibited good inhibitory effects against *F. oxysporum,* showing superiority over the commercial fungicide hymexazol. Among them, compound **80** completely inhibited *F. oxysporum* growth, offering the lowest EC_50_ value of 29.89 µg mL^−1^ (98.60 µM).

**Scheme 48:**
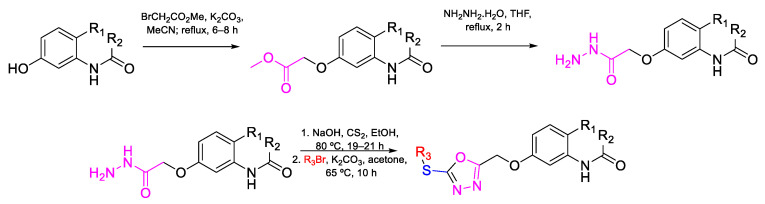
Linear synthesis of 1,3,4-oxadiazoles reported by Wang.

Wang et al. (2020) obtained *N*-(2-alkyl-5-((5-(alkylthio)-1,3,4-oxadiazol-2-yl)methoxy)phenyl)amide-type compounds. The synthetic steps are shown in Scheme 48 [113]. Under reflux conditions, 7-hydroxy-3,4-dihydroquinolin-2(1*H*)-one/*N*-(3-hydroxyphenyl)acetamide was reacted BrCH_2_CO_2_CH_3_ to obtain the respective methyl esters. Secondly, they were reacted with hydrazine hydrate using THF as a solvent affording the respective hydrazides. The reaction between hydrazides, CS_2_ under basic conditions, and the subsequent reaction against alkyl bromides allowed to obtain target compounds with moderate to high yields (Scheme 48).

**Scheme 49:**
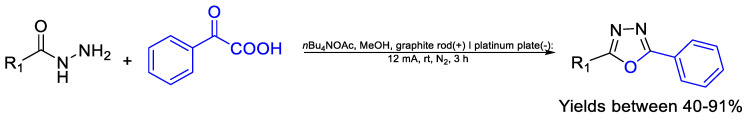
Electrochemical synthesis of 2,5-disubstituted 1,3,4-oxadiazoles.

Lu et al. (2020) performed intermolecular electrochemical cyclization between α-keto acids and acylhydrazines to synthesize 2,5-disubstituted 1,3,4-oxadiazoles [114]. This transformation can be carried out under mild reaction conditions without external oxidants, bases, and transition metal catalysts. Both symmetrical and unsymmetrical 2,5-disubstituted 1,3,4-oxadiazoles could be prepared according to the excellent choice of the substrates. The reaction of benzohydrazide and 2-oxo-2-phenylacetic acid afforded the desired products in 88% yield, which meant nearly no yield loss occurred than the reaction of 0.5 mmol scales (Scheme 49).

**Scheme 50:**
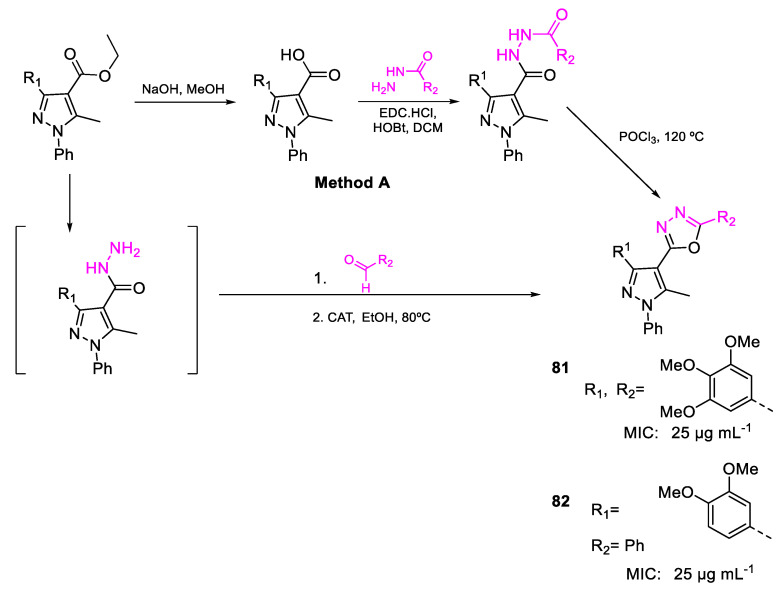
Synthesis of active molecules **81** and **82**.

Ningaiah et al. (2014), based on literature studies that reveal that linked biheterocyclic compounds containing pyrazole and pyrazolyl-1,3,4-oxadiazole analogs possess biological activity, synthesized some heterocycles with a 1,3,4-oxadiazole moiety attached to the pyrazole ring [115]. The synthesis of 1,3,4-oxadiazole derivatives of pyrazole employed two synthesis strategies A and B, which start from ethyl 5-methyl-1,3-diphenyl-1*H*-pyrazole-4-carboxylate as a precursor. The sequence of two reactions (method A and B) for the preparation of biheterocycle 2-(5-methyl-1,3-diphenyl-1*H*-pyrazol-4-yl)-5-phenyl-1,3,4-oxadiazoles occurred using the key intermediates: 5-methyl-1,3-diphenyl-1*H*-pyrazole-4-carboxylic acid and 5-methyl-1,3-diphenyl-1*H*-pyrazole-4-carbohydrazides (Scheme 50). The biological activity results demonstrated that the compounds containing the group -NO_2_ and -OCH_3_ in the phenyl ring, which in turn is attached to the C-3 of the pyrazole residue, have good antibacterial activity. Regarding *FOX*, the compound with the highest antifungal activity corresponds to **81** and **82**, characterized by aromatic rings linked to the pyrazole and oxadiazole heterocyclic systems with at least two methoxyl groups.

**Scheme 51:**
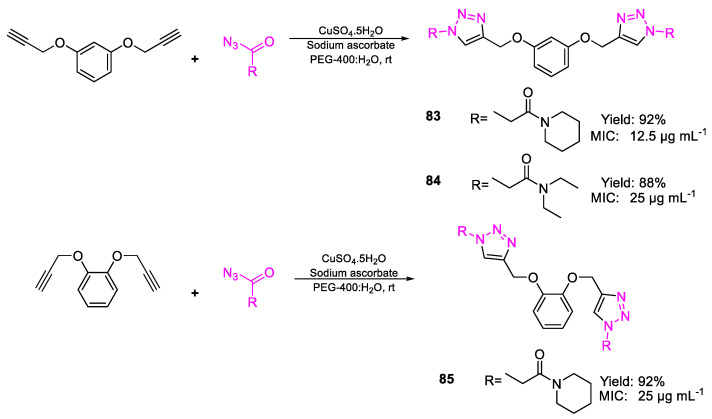
Synthesis of active molecules **83**–**85**.

Azole compounds of the type 1,2,3-triazoles attract more attention as they have excellent biological and physical properties [116,117]. These heterocycles can be obtained by the copper (I) catalyzed 1,3-dipolar cycloaddition reaction of azide and alkyne, as shown in previous reports. Deshmukh et al. (2019) carried out the design and synthesis of 1,2,3-dimeric triazoles from azides and *bis*(prop-2-yn-1-yloxy)benzene type compounds using 1,3-dipolar cycloaddition with yields of 80 to 90% (Scheme 51). All the synthesized compounds were analyzed for their antifungal activity against *F. oxysporum* (NCIM 1332). Compound **83** was better than the miconazole positive control (MIC 25 µg/mL) to inhibit this pathogenic fungus with a MIC value of 12.5 µg/mL. In contrast, compounds **84** and **85** showed the same activity as miconazole [118].

**Scheme 52:**
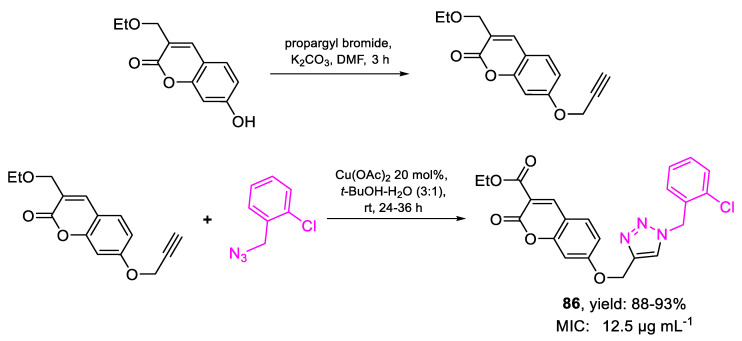
Synthesis of active molecule **86**.

Shaikh et al. (2016) also synthesized through the click chemistry approach a series of new ethyl 7-((1-(benzyl)-1*H*-1,2,3-triazol-4-yl)methoxy)-2-oxo-2*H*-chromen-3-carboxylates (Scheme 52) with yield percentages of 80 to 90% (Scheme 52). The compounds contained in their structure a coumarin nucleus and 1,2,3-triazole as the only molecular scaffolds were evaluated at the in vitro level against *FOX* to determine the effect of the structural transformation in the 7-hydroxycoumarin derivative. Compound **86** (Cl group in ortho position) was the one that presented the lowest MIC value (12.5 µg/mL) compared to miconazole (MIC 25 µg/mL) used as a positive control. The computational studies carried out (molecular docking and ADME prediction) supported the results obtained from the in vitro tests. They presented high affinity and ADME properties similar to those of a drug [119].

**Scheme 53:**
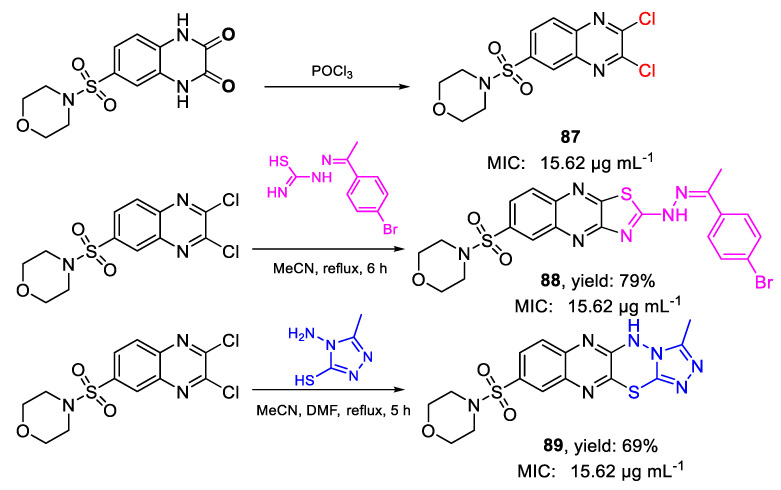
Synthesis of active molecules **87**–**89**.

Quinoxaline is one of the essential classes of heterocycles since it is present in biologically and pharmacologically active compounds to act as antiallergics, antidepressants, anxiolytics, and antimicrobials [120,121,122]. Ammar et al. (2020) designed and synthesized a series of thiadiazino[5,6-*b*]quinoxaline and thiazolo[4,5-*b*] quinoxaline derivatives from the reaction of 2,3-dichloro-6-(morpholinosulfonyl)quinoxaline with thiosemicarbazide or thiocarbohydrazide and thiourea derivatives (Scheme 53). All products were evaluated against *F. oxysporum* (RCMB 008002), being compounds **87**–**89** that presented lower MIC values (15.62 µg/mL) compared to amphotericin B (MIC 31.25 µg/mL). These results showed that the antifungal activity is significantly influenced by the structure and the different substituents on the quinoxaline ring [123].

Although there have been relatively few benzothiazoles found in nature compared to other types of heterocyclic motifs, they play a remarkable role in current medicinal chemistry. The inherent affinity of benzothiazole derivatives for diverse biological receptors makes them an ideal source for further developing lead candidates. This versatility has continually attracted academic attention, consequently disclosing a broad spectrum of bioactivities that benzothiazole analogs possess, such as analgesic, anticancer, anticonvulsant, antidiabetic, anti-inflammatory, antimicrobial, antimalarial, antiviral, and fungicidal [124,125,126]. Recently, Ballari et al., inspired by the properties of this privileged heterocyclic scaffold, reported in 2017 the synthesis of a series of 2-benzyl mercaptobenzoazoles through a green SN_2_ methodology between the available 2-mercaptobenzothiazole and 2-mercaptobenzoxazole with different benzyl halides as starting materials [127]. The entire series was easily synthesized in excellent yields between 71% and 93% and evaluated for their antifungal activity. Most compounds bore an antifungal activity similar to or better than the reference drug Captan against FOX M15-Pa. Remarkably, derivatives **90** and **91** (Table 1) were more than 200 and 7000 times active than Captan against this phytopathogen, exhibiting the excellent IC_50_ values of 0.0067 µM and 0.23 µM, respectively. Furthermore, all else being equal (with just one clear exception), the benzothiazole series tended to be more active against this mold than the benzoxazole series.

Ballari et al. synthesized a series of 2-benzylsulfonyl benzothiazoles [128]. They were interested in the antifungal activity differences between these oxidized derivatives and the earlier reported non-oxidized compounds. Thus, a one-pot substitution/oxidation sequence in an aqueous medium allowed the obtention of the sulfonyl products with excellent yields (75–89%). Compounds **92**–**94** (Table 1) showed good inhibitory activity against *FOX*. Moreover, compound **92** exhibited a remarkable IC_50_ value of 2.3 µM, 34 fold more active than the 2-((4-methylbenzyl)thio)benzo[*d*]thiazole analog. However, an evident tendency was not established after pairing all the previous non-oxidized benzothiazole series with the oxidized counterpart. Bondock et al., on the other hand, followed an approach of hybrid molecules incorporating the benzothiazole nucleus in the search for new lead candidates as antimicrobial agents. They described the synthesis of two groups of hybrid compounds comprising the benzothiazole moiety in conjunction with either pyrazole or pyrimidine heterocycles [129]. Most compounds exhibited some degree of inhibitory activity against a *FOX* strain. Among them, derivatives **95**–**97** were the most actives. Later in 2010, Bondock et al. reported synthesizing another hybrid analog series around the benzothiazole scaffold in conformity with their earlier work [130]. They employed the same cyanoacetamide group as the linker and precursor for constructing the different thiophene, pyrazole, and thiazole heterocycle rings. Compounds **98** and **99** (Table 1) showed good inhibitory activity against *FOX* after screening 14 of the total synthesized compounds for their antimicrobial activity. The thiophene-bearing derivative achieved a close antifungal activity with the reference drug cycloheximide.

**Scheme 54:**
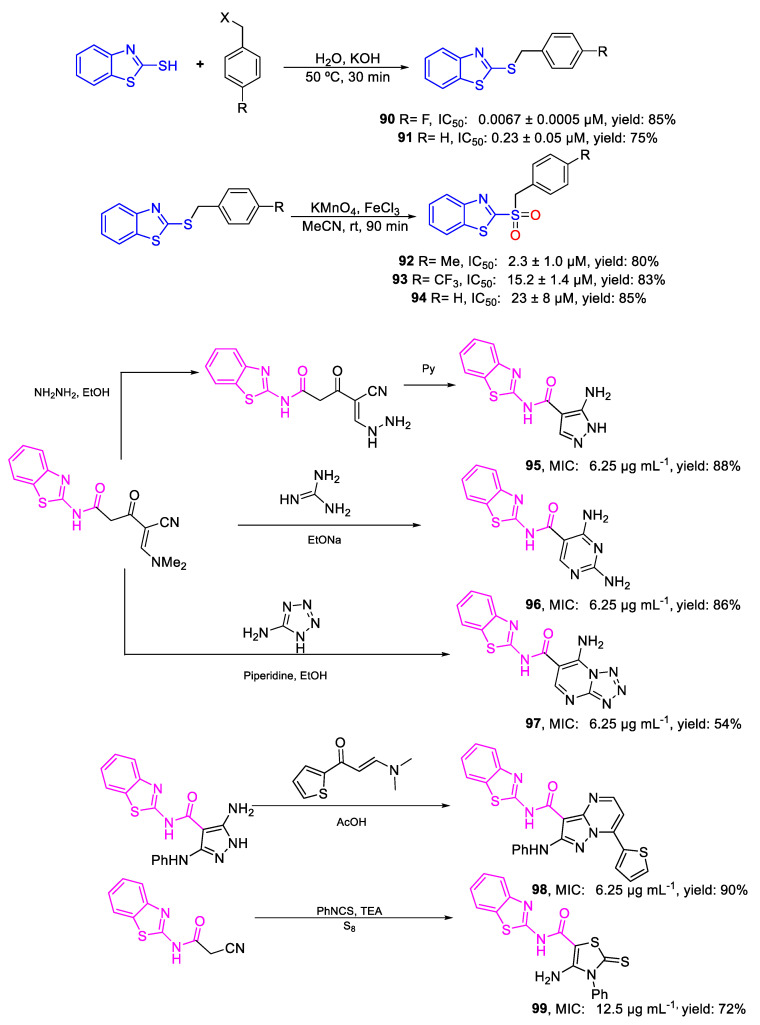
Synthesis of active molecules **90**–**99**.

In contrast, analogs bearing the thiazoline and pyrazole moieties were 3 and 4 times less active, respectively, compared with cycloheximide. All the compounds were synthesized through a linear strategy with *N*-(benzothiazol-2-yl)-2-cyanoacetamide as the starting material (Scheme 54). Thus, using a modified Gewald reaction catalyzed with triethylamine, the thiazoline was afforded 72% yield after heating a mixture containing elemental sulfur, phenyl isothiocyanate, and the amide.

**Scheme 55:**
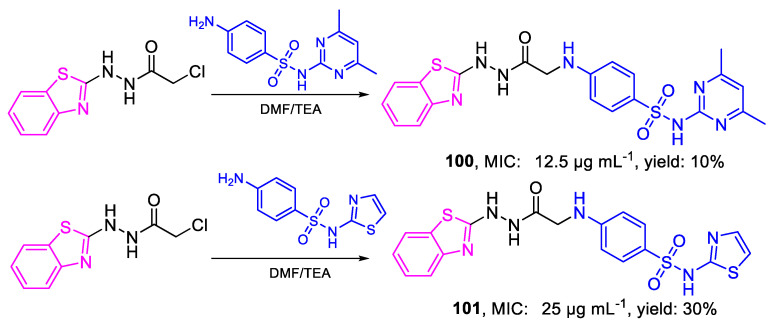
Synthesis of active molecules **100** and **101**.

Fadda et al. (2019) reported another set of benzothiazole analogs following the same narrative of screening hybrid compounds to inhibit some microorganisms [131]. They described the synthesis through a linear sequence of some novel derivatives involving pyrimidine, thiazole, and phthalimide rings linked to the benzothiazole core. Additionally, various fused benzothiazolopyridine compounds were also synthesized (Scheme 55). Among all the tested products, only compounds **100** and **101** displayed a noticeable antifungal behavior against *FOX*. Pyrimidine derivative presented almost half the inhibition activity (MIC 25.85 µM) regarding the reference drug cycloheximide (MIC 11.10 µM), while thiazole derivative was five times lesser active (MIC 54.28 µM). These results disclosed the crucial role that plays the sulfonamide group in the inhibition of this probed organism. The active molecules reported in all the works previously discussed reiteratively reinforce the relevance of this structural feature.

The hydrazone linkage has been applied successfully by Zha et al. (2017) in their synthesized derivatives, some of them performing a good *in vitro* antifungal behavior [132]. Hence, it demonstrates that this molecular feature comprises a valuable and practical tool for bonding the benzothiazole core to a wide range of structural motifs without a noticeable cost in the bioactivity response. This set of benzothiazole analogs strongly resembles topologically the previously discussed Ballari’s works. However, Zha et al. followed a contrasting approach. They commenced from *o*-toluidine to construct the benzothiazole nucleus bearing a methyl in the 7th position, consisting of a distinct substitution pattern among all the examples reviewed. Thus, all hydrazone products were afforded in excellent yields (78–92%) after the treatment of several aldehydes with 2-hydrazinyl-7-methylbenzo[*d*]thiazole under a catalytic amount of acetic acid. In turn, the methylbenzothiazole template was prepared by the Hugerschoff reaction conditions, involving the oxidative cyclization of the 1-(*o*-tolyl)thiourea with bromine. Recently, several different methodologies have been reported for the assembling of substituted aminobenzothiazoles from thioureas. Some examples include the employment of alternative bromine sources, metal-free base-promoted cyclization, palladium-catalyzed intramolecular cyclization, palladium C-H functionalization with intramolecular C-S formation, cooper or iron-catalyzed one-pot tandem reactions, among others.

**Scheme 56:**
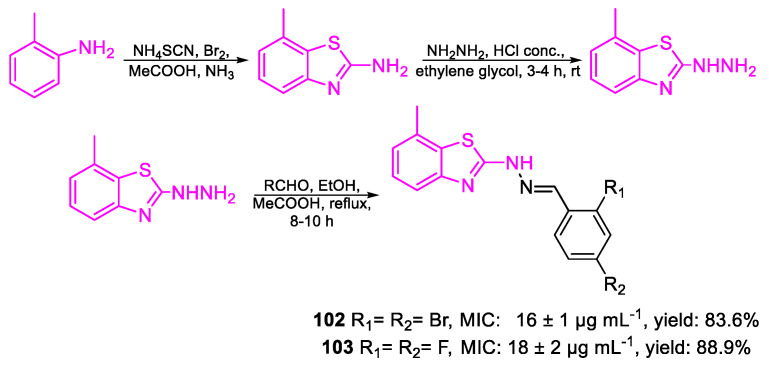
Synthesis of active molecules **102** and **103**.

Regarding the antifungal evaluation, the dihalogenated compounds **102** and **103** displayed the best in vitro inhibitory activity against *FOX* (Scheme 56). Other derivatives also showed good activity comparable to the standard drug ketoconazole (45.16 µM). The SAR study pointed out that electron-withdrawing groups (NO_2_, Cl, Br, F) at *ortho* and *para* positions tend to enhance the bioactivity. On the other hand, electron-donating groups like -OH and -OCH_3_ altogether with substituents at meta position tend to be detrimental to the inhibitory behavior.

The examples mentioned above reinforce the importance of the benzothiazole scaffold in searching for new lead candidates against this problematic phytopathogen. Although diverse variations have been performed and screened, several structural features have not been thoroughly studied. One of the main aspects that have been relegated is the role of substituents around the benzothiazole core. Only variations at the 2nd position have been widely explored, possibly due to synthetic convenience. The reported cases of compounds with different substitution patterns that presented an excellent inhibitory response against other fungi illustrate the importance of exploring new derivatives following this approach. However, most studies do not implement systematic structural variations that lead to more accessible interpretations and comprehension.

**Scheme 57:**
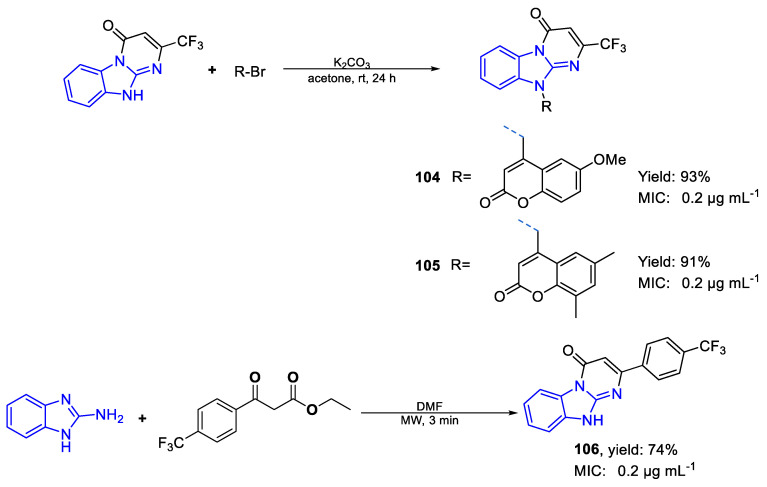
Synthesis of active molecules **104–106**.

Puttaraju (2013) published the synthesis of 10-((2-oxo-2*H*-chromen-4-yl)methyl)-2-(trifluoromethyl)benzo[4,5]imidazo[1,2-*a*]pyrimidin-4(10*H*)-one, which was carried out by reaction of 4-bromomethyl coumarins with dihydrobenzo[4,5]imidazo[1,2-*a*]pyrimidin-4-ones in the presence of anhydrous K_2_CO_3_ in dry acetone at room temperature for 24 h (Scheme 57). The heterocyclic precursor system can be obtained from the cyclo condensation between 2-aminobenzimidazole and the respective ketoester. The minimum inhibitory concentrations (MIC) were determined by the serial dilution method. The compounds **104** and **105** were highly active against *F. oxysporum* with a MIC of 0.2 μg/mL [133].

**Scheme 58:**
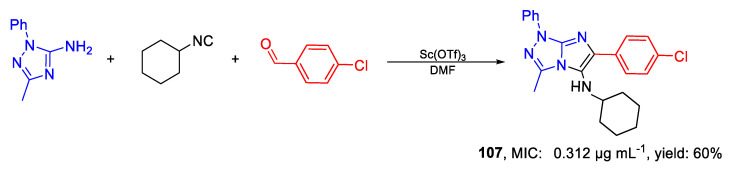
Synthesis of active molecule **107**.

Aouali et al. (2015) reported the synthesis of imidazo[2,1-*c*][1,2,4]triazoles through a Groebke-type multicomponent reaction between 5-amino-1,2,4-triazoles, aromatic aldehydes, and alkylisonitrile. The heterocyclic system was formed in DMF at 80 °C, in a reaction that proceeds via the formation of an iminium species followed by a [4+1] cycloaddition with the isonitrile using scandium triflate as a Lewis acid catalyst (Scheme 58). The synthesized imidazo[2,1-*c*][1,2,4]triazole derivatives were screened for antibacterial, antifungal, and antioxidant activities. Among the tested compounds, **107** showed potent antibacterial and antifungal activities [134].

**Scheme 59:**
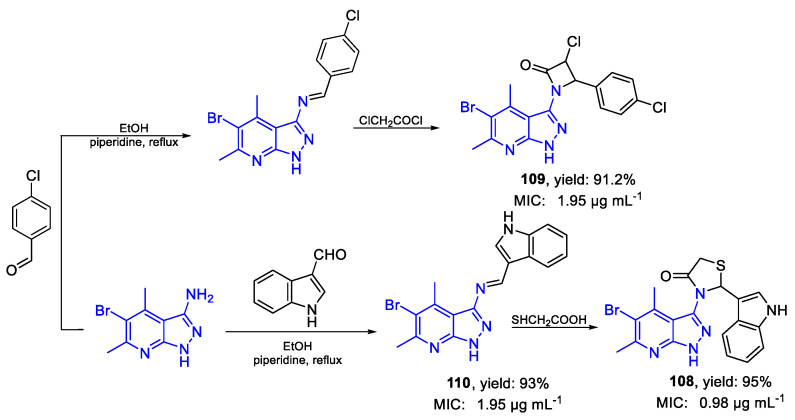
Synthesis of active molecules **108**–**110**.

Salem and Ali (2016) obtained several pyridine derivatives from Schiff’s bases by intermolecular cyclization (Scheme 59). The reaction of some Schiff’s bases with chloroacetyl chloride in dioxane containing triethylamine afforded tetrahydrocarbazolyl azetidinones **109**. The reaction of heteroaromatic Schiff’s bases **110** against 2-mercaptoacetic acid afforded compound **108**. Compounds **108–110** exhibited better antimicrobial activity against *Salmonella typhimurium*, *Aspergillus niger*, and FOX. In most cases, the inhibitory potency exhibited by the tested compounds is lower than that of the standard antimicrobial agents. A noteworthy exception arises with compound **108**, nearly as active as the standard antimicrobial drug Amphotericin B against FOX [135].

**Scheme 60:**
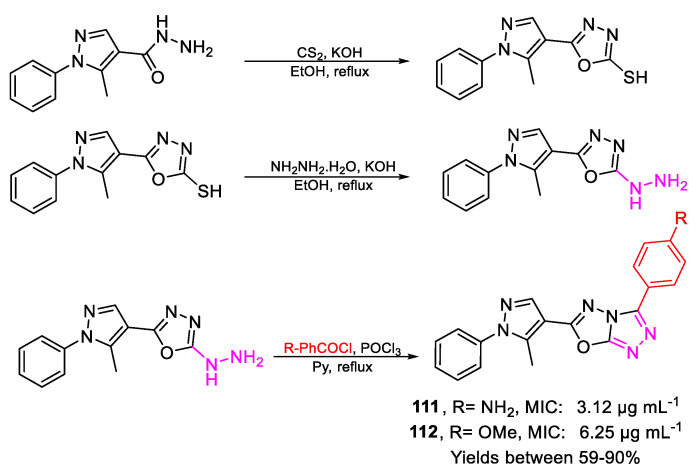
Synthesis of active molecules **111** and **112**.

The use of hybrid heterocyclic systems, also called a fusion of hetero systems in a single molecular framework, has received considerable attention. The biological activity results demonstrated for heterocyclic systems of pyrazole, oxadiazole, and triazole keep current research searching for new bioactive molecules. Kumar et al. (2018) reported the synthesis of 3-aryl/hetaryl-6-(5-methyl-1-phenyl-1*H*-pyrazol-4-yl)-[1,2,4]triazolo[3,4-*b*][1,3,4]oxadiazoles. The synthesis, described as three sequential steps, begins with forming a 1,3,4-oxadiazole heterocyclic system by reacting cyclo condensation with carbon disulfide. Subsequently, the thiol group is replaced by a hydrazine group, which finally reacted with an aryl acyl chloride in POCl_3_, leading to the formation of compounds **111** and **112** (Scheme 60). The structure–activity relationship for the tested compounds compared with the standard drug Itraconazole reveals that the compounds in which triazole moiety 4-aminophenyl **111** and 4-methoxyphenyl **112** exhibited prominent antifungal activity, nearly equal to the Itrazole standard against all the tested fungi [136].

**Scheme 61:**
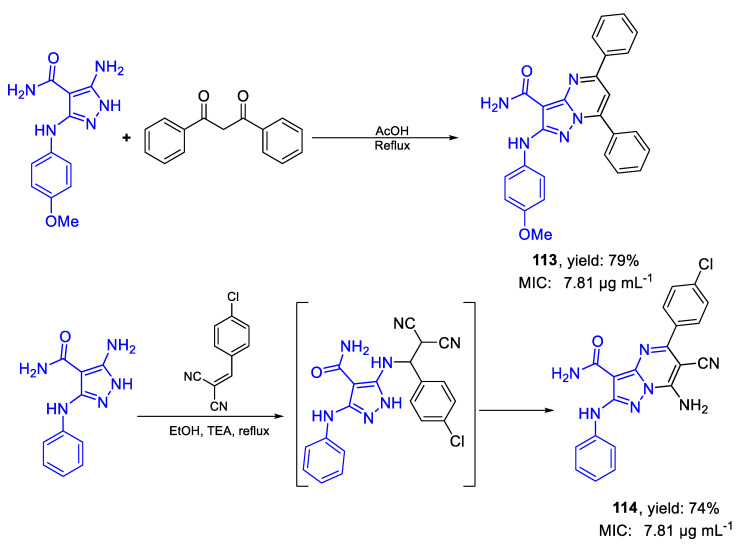
Synthesis of active molecules **113** and **114**.

Pyrazolo[1,5-*a*]pyrimidines have shown promissory biological and medicinal applications. Hassan (2017) reported the synthesis of novel 2-((4-methoxyphenyl)amino)-5,7-diphenylpyrazolo[1,5-*a*]pyrimidine-3-carboxamide type compounds from 1,3-dicarbonyl compounds and 5-amino-3-(phenylamino)-1*H*-pyrazole-4-carboxamide using a cyclo condensation reaction in acid media. Using 1,2-unsaturated malononitrile derivatives and 5-amino-3-(phenylamino)-1*H*-pyrazole-4-carboxamide, the cyclo condensation in basic media afforded 7-amino-5-(4-chlorophenyl)-6-cyano-2-(phenylamino)pyrazolo[1,5-*a*]pyrimidine-3-carboxamide in high yield (Scheme 61). Antifungal activity experiments allowed to establish that the absence of the methoxy group (*p*-OCH3) in the para position on the phenyl ring tends to higher antimicrobial activity. Some derivatives bearing the 4-chlorophenyl group were more active than those bearing the 4-fluorophenyl group and those bearing the phenyl group [137].

**Scheme 62:**
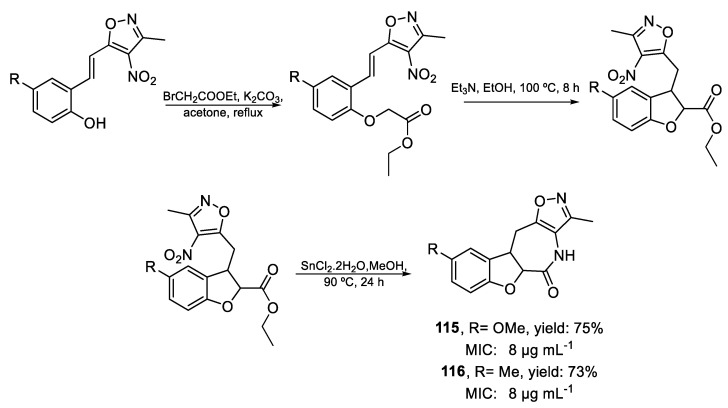
Synthesis of active molecules **115** and **116**.

Rajanarendar et al. (2013) obtained novel series of dihydro benzofuro[3,2-*e*]isoxazolo[4,5-*b*]azepin-5(5*aH*)-ones from 3,5-dimethyl-4-nitroisoxazole. Initially, 3,5-dimethyl-4-nitroisoxazole reacted with substituted salicylaldehydes forming Schiff bases. Then, Schiff bases reacted with ethyl bromoacetate suffering alkylation on the phenolic hydroxyl group and adding a side chain with ester functionality with acidic hydrogens in position 2 concerning the carboxylate group. The addition of TEA favors the formation of an enolate ion that promotes an annulation reaction towards a dihydro benzofuran derivative, which will finally undergo a reduction in the nitro group attached to the isoxazole ring, forming a nucleophilic amino group that attacks the ester group and promotes a 7*-exo-trig* cyclization towards the formation of the azepine-5-one ring (Scheme 62). The antifungal activity data (Table 1) revealed that compounds **115** and **116** exhibited high antifungal activity by inhibiting the growth of fungi to a remarkable extent compared to standard drug fluconazole, which may be due to methyl methoxy substituents on the benzene ring. However, the degree of spore germination inhibition varied with the test compound and the fungi under investigation [138].

**Scheme 63:**
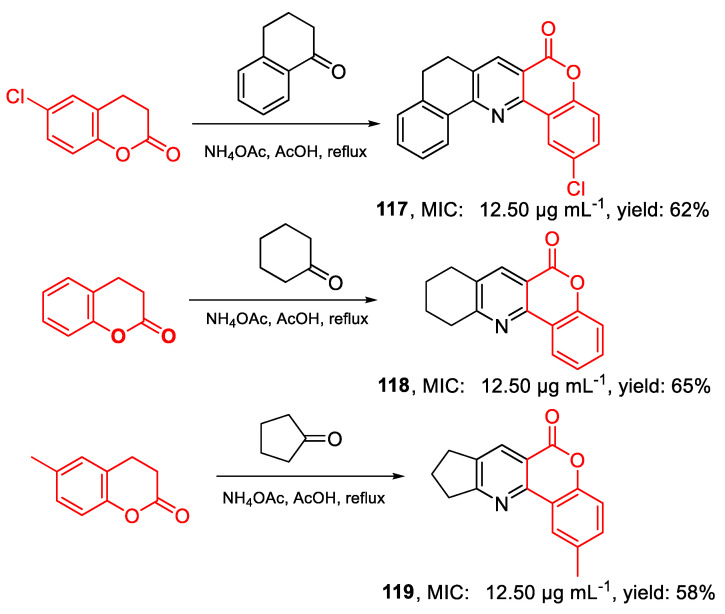
Synthesis of active molecules **117**–**119**.

Patel et al. (2012) studied the reaction of 4-chloro-2-oxo-2*H*-chromene-3-carbaldehydes against cyclopentanone, cyclohexanone, or a-tetralone in the presence of ammonium acetate in refluxing glacial acetic acid affording fused chromenone derivatives with moderate yields (Scheme 63). Compounds **117**–**119** showed good MIC for *FOX* (ATCC 16417) [139].

**Scheme 64:**
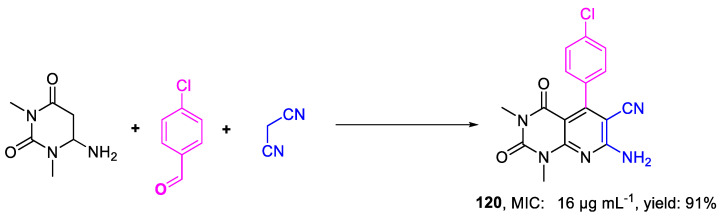
Synthesis of active molecule **120**.

Aryan et al. (2019) published the synthesis of highly substituted pyrido[2,3-*d*]pyrimidine derivatives. These compounds were obtained by the reaction of 4-chlorobenzaldehyde, malononitrile, and 4- or 6-aminouracil using choline chloride/urea (1:2) mixture as a deep eutectic solvent with high yields (Scheme 64). Most of the pyrido[2,3-*d*]pyrimidine derivatives inhibited *Aspergillus fumigatus* (PTCC 5009) and *Candida albicans* (PTCC 5027). At the same time, some derivatives showed an inhibitory effect on *FOX* (PTCC 5115), similar to the two standard antifungal agents, ketoconazole and nystatin (Table 1). The most promissory results were recorded for product **120**, which has a chlorine atom attached to position 7 in the phenyl ring [140].

**Scheme 65:**
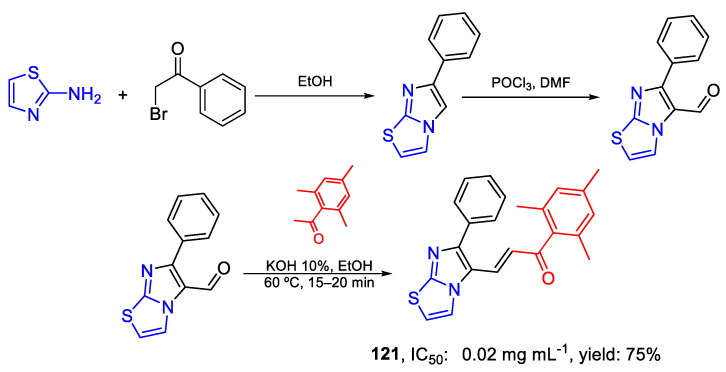
Synthesis of active molecule **121**.

Koudad et al. (2019) reported the synthesis of imidazothiazole derivatives. These compounds were synthesized via Claisen–Schmidt condensation between functionalized aldehydes and different methyl ketones. The imidazo[2,1-*b*]thiazole carbaldehyde type compounds were prepared using a linear strategy where the first step was the reaction of thiazol-2-amine and 2-bromoacetophenones. The reagents experimented with an initial *N*-alkylation and subsequent cyclo condensation to the fused heterocyclic system of imidazo[2,1-*b*]thiazole. The obtention of imidazo[2,1-*b*]thiazole carbaldehyde type compounds occurred by formylation with DMF in the presence of POCl_3_ (Scheme 65). The antifungal test of the five imidazothiazole derivatives experienced at five different doses acted differently on the mycelia *FOX*. Indeed, mycelial growth is completely inhibited by compound **121** with IC_50_ not exceeding 0.07 mg/mL [141].

**Scheme 66:**
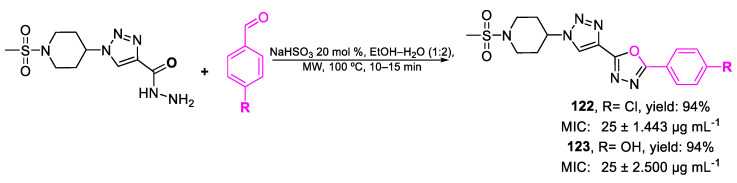
Synthesis of active molecules **122** and **123**.

Sangshetti (2011) performed the synthesis of 2,5-disubstituted 1,3,4-oxadiazole, a heterocycles class that has attracted significant interest in medicinal chemistry. The synthesis of these compounds was carried out by cyclo condensation reaction between 1*H*-1,2,3-triazole-4-carbohydrazide and aromatic aldehydes. The yields of the obtained compounds were between 90–95% using microwave and 87–91% using a one-pot reaction of hydrazide, aromatic aldehyde in ethanol: water using sodium bisulfite as a catalyst (Scheme 66). The synthesized compounds were found to show good antifungal activity. From the antifungal activity data (Table 1), it is observed that compounds **122** and **123** are the most active among all tested compounds against *FOX*s [142].

**Scheme 67:**
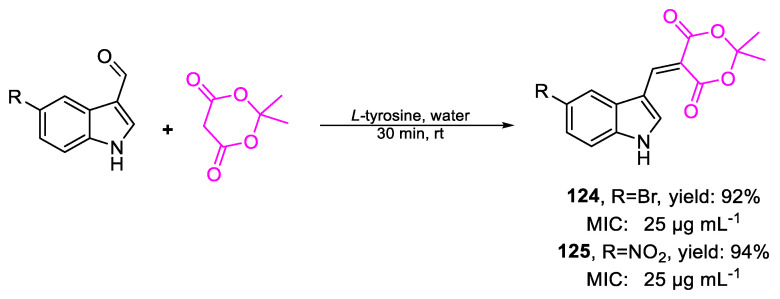
Synthesis of active molecules **124** and **125**.

Thirupathi et al. (2014) studied the condensation of substituted indole-3-aldehydes with Meldrum’s acid to obtain compounds **124** and **125**, respectively. The reactions were performed in water at room temperature for 30 min using *L*-tyrosine as a catalyst leading to the formation of 5-((1*H*-indol-3-yl)methylene)-2,2-dimethyl-1,3-dioxane-4,6-dione derivatives (Scheme 67). This method was applied to a wide range of indole-3-carboxaldehyde, including *N*-substituted-indole-3-carboxaldehyde. The compounds **124** and **125** were tested and showed excellent antifungal activity against *Rhizoctonia solani* and *FOX* [143].

**Scheme 68:**
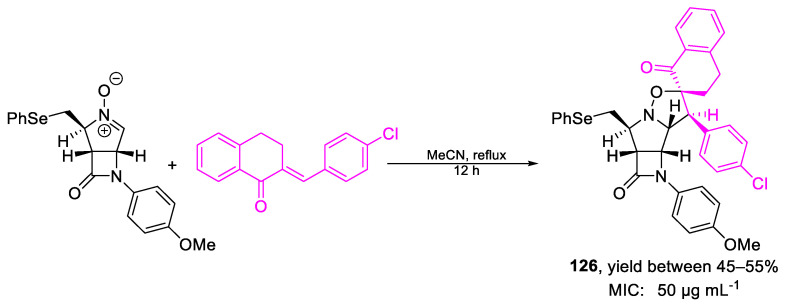
Synthesis of active molecule **126**.

Arumugam et al. (2010) synthesized novel lactams fused to spiroisoxazolidine chromanones and tetralones ring systems. The desired compounds were obtained by intermolecular 1,3-dipolar cycloaddition reaction of bicyclic nitrone with unusual dipolarophiles, arylidene chromanones/tetralones (Scheme 68). The synthesized compound **126** effectively controlled both fungal pathogens, namely FOX and *M. phaseolina,* with MIC values of 50 mg/mL (Table 1) [144].

**Scheme 69:**
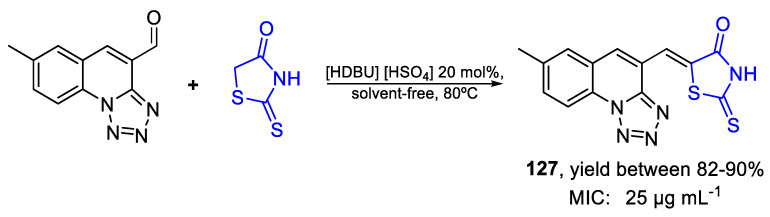
Synthesis of active molecule **127**.

Subhedar et al. (2016) described the protocol to synthesize novel arylidene-rhodanine systems known as attractive targets in medical and organic, and medicinal chemistry. The synthetic protocol uses [HDBU] [HSO_4_] as a catalyst in the Knoevenagel condensation between 7-methyltetrazolo[1,5-*a*]quinoline-4-carbaldehyde and 2-thioxothiazolidin-4-one under solvent-free conditions (Scheme 69). The optimized reaction conditions were 20% by mole of the catalyst [HDBU] [HSO_4_], a temperature of 80 °C and a solvent-free condition, which led to the synthesis of the highly substituted tetrazoloquinolidine-rhodanine conjugates from the corresponding tetrazoloquinoline aldehyde and rhodanine derivatives with good to excellent yields (82–90%). The compound **127** was the most active against FOX, showing a MIC value of 25 μg/mL [145].

**Scheme 70:**
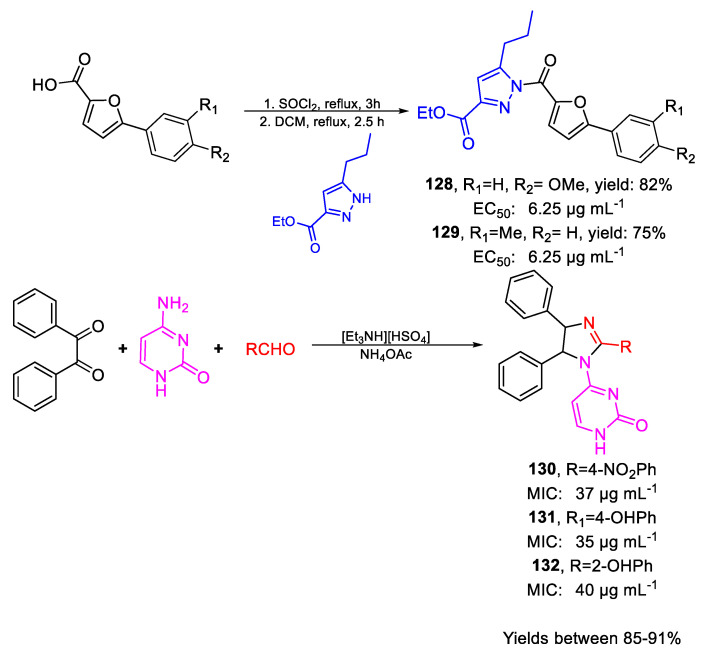
Synthesis of active molecules **128**–**132**.

Ahmed et al. (2019) designed and synthesized a series of new pyrazole derivatives containing 5-phenyl-2-furan by amidation reaction between furan carboxylic acid derivatives and 1*H*-pyrazole assisted by thionyl chloride with good yields (Scheme 70). The most active compounds against *FOX* were **128** and **129**; however, all the evaluated compounds showed lower activity than pentiopyrad, pyrimorph, and hymexazole [146]. Recently, Tiwari et al. (2018) reported on the synthesis of a new series of derivatives of 4-[(4,5-diphenyl-2-phenyl/2-substituted heteryl)-1*H*-imidazol-1-yl] pyrimidine-2(1*H*)—one using a protocol that employed a cyclo condensation in the ionic liquid triethylammonium hydrogen sulfate [Et_3_NH] [HSO_4_]. Using benzyl, 4-aminopyrimidin-2(1-*H*)-one, and various aldehydes as starting reagents, the reactions were completed in approximately 25 to 35 min, and the yields of the newly synthesized compounds were in the range of 85 to 91%. The screening study of the antifungal activity of the synthesized compounds reveals that almost all derivatives have shown excellent antifungal activity. Furthermore, the ergosterol extraction and quantification assay method and the coupling study indicate that the synthesized compounds **130**–**132** act by inhibiting ergosterol biosynthesis by inhibiting the enzyme lanosterol 14α-demethylase [147].

## 4. Conclusions and Perspectives

This review allowed us to establish that most of the synthetic methods published for promising antifungal agents usually employ cyclo condensation reactions. These protocols generally tend to have moderate to good yields, which depend on the structural nature of the precursors, the reaction conditions, and the use of catalysts. Among the most used catalysts, the use of porous materials, composites that can cause acidic or basic catalysis, have recently increased, although both inorganic and organic acids and bases are still being used. In addition, numerous protocols evidenced the use of metal catalysts, which tend to improve performance and selectivity under mild conditions. Regarding the energy sources used, although the use of conventional heating is maintained, many methodologies have more frequently used microwave or ultrasound irradiation to achieve better performance. On the other hand, the manuscripts cited and discussed in this review clearly showed that heterocyclic compounds play an essential role in controlling a phytopathogen such as *FOX*, being benzothiazole derivatives, the most studied compounds with the highest antifungal activity (Table 1). Some reports have described biological and environmental effects and their potential activity, degradation pathways, and subproducts characterization of synthetic heterocycles such as podophyllotoxin derivatives [148], rhodamine derivatives and analogs [149], benzothiazole and benzotriazole derivatives—which have emerged as contaminants in aquatic environments and toxic to aquatic organisms [150,151,152,153]—and polycyclic (hetero)aromatic hydrocarbons compounds which recently was demonstrated their predominance in contaminated food samples and their relationship with potential toxicity [154]. However, further studies are necessary to establish these promissory antifungal agents’ potential cytotoxicity and environmental risks.

Future research on this type of heterocyclic compounds could give more promising results in agrochemistry. It is hoped that this information will lead to the design of better molecules with improved antifungal properties and greater specificity as the development of new synthetic strategies. However, there is an urgent need to direct research related to the synthesis and design of new bioactive molecules against *FOX*, considering the described antecedents in this review. The design of novel antifungal agents against *FOX* should be oriented to inhibit specific enzymes, commonly called molecular targets. Thus, Catharina and Carels (2018) performed a systematic identification of specific enzymes for *FOX* [155]. In addition, they described the characterization of enzymatic functionalities associated with protein targets that could be considered for the control of root rots induced by *FOX* such as chitin synthase [156], UDP-N-acetylglucosamine diphosphorylase [157], the decapping scavenger enzyme (DcpS, m7GpppX diphosphatase) [158], carnitine acetyltransferase [159], hydroxyanthranilate 3,4-dioxygenase [160,161], ureidoglycolate lyase [162], and holocytochrome-c synthase (HCCS, also known as cytochrome c heme lyase) [163]. It is necessary to focus on vital processes such as cell membrane stability, respiration, mitosis and cell division, and signal transduction. The cell membrane performs many biological functions: to prevent the entry of large molecules, provide the cell’s shape, maintain the water potentials in the cell, and participate in signal transduction. It has been established as adverse effects of fungicides, affecting the membrane of microorganisms, which alter their structure and function [164]. Azole fungicides, such as triazoles, interrupt the biosynthesis of ergosterol, an essential sterol of fungal cell membranes, by inhibiting cytochrome P450 eburicol 14α-demethylase (CYP51). This inhibition prevents the demethylation of eburicol, the primary substrate of CYP51 in most filamentous fungi such as *FOX*, which leads to a depletion of ergosterol and an accumulation of non-functional 14α-methylated sterols [165]. Inhibition of this enzyme could deplete ergosterol and changes the fluidity of the membrane in the lipid bilayer, which leads to a reduction in the activity of crucial membrane enzymes and, if ergosterol levels are low enough, blocks the “sparking” reaction necessary for the re-initiation of fungus growth [166]. Fungicides that alter cell division processes presumably affect β-tubulin, since these can inhibit the assembly of α- and β-tubulin heterodimers in microtubules, which are vital for various processes such as signaling motility, division cell, and mitosis [167,168]. Moreover, fungicides that become inhibitors of this metabolic process can bind to cytochrome b [169], an enzyme that is part of the bc1 complex, which is present in the internal mitochondrial membrane of eukaryotic organisms and is responsible for catalyzing the transfer of electrons from ubiquinol to cytochrome c [170]. Compounds that inhibit mitochondrial respiration block the electron transfer process in the airway and lead to an energy deficit due to a shortage of ATP [171]. Many of the fungicides can cause damage to process such as DNA replication and transcription in phytopathogenic fungi. Within the enzymes that involve these metabolic processes, topoisomerases are ubiquitous enzymes found in various living organisms, including fungi pathogens [172], as they are necessary for the maintenance of DNA topology [173]. The main goal to direct the design of novel bioactive compounds is the molecular characterization of these enzymatic targets and the determination of their quaternary structure, their active site and mainly, successful protocols for their obtention. However, few reports have been published for *FOX* enzymatic targets making difficult the access to this information.

Despite this, computational tools such as molecular docking, molecular dynamics, and quantitative structure–activity relationship (QSAR) offer an essential alternative for the rational design of new antifungal agents against *FOX*. Recently, in silico molecular docking studies of pyrrolo(1,2-*a*)pyrazine-1,4-diones, hexahydro, and pyrrolo(1,2-*a*)pyrazine-1,4-diones, hexahydro-3(2-methylpropyl)pyrrolo(1,2-*a*)pyrazine-1,4-diones against some enzymatic targets, showed the potential of these compounds as bioactive multitargeting compounds [174]. Hydrazone derivatives bearing imidazole or benzimidazole nucleus were designed, synthesized, and evaluated for their antioxidant, antifungal, and anti-acetylcholinesterase activities. Molecular docking studies of the most active compounds showed reasonable binding modes in the active site of *FOX* FGB1 enzyme and acetylcholinesterase, and in silico predictions of ADME and pharmacokinetic parameters indicated that these compounds should have good oral bioavailability [175]. The structure–antifungal activity relationship studies of fusarubin analogs using molecular docking and simulations-allowed establishing these compounds’ possible mechanism against three target enzymes [176]. Finally, molecular docking studies of some Schiff bases derived from 5-(morpholinosulfonyl)indol-2,3-dione and appropriate amines or hydrazide derivatives indicated good binding with the evaluated enzymatic targets lower binding energy of the most promising compounds than a standard drug used [177]. These recent antecedents involving computational tools leading to the generation of structure–activity correlation models will allow the effective obtaining of new agrochemical agents.

## Figures and Tables

**Table 1:** Compendium of the most promising organic compounds against several *FOX* synthesized between 2010–2020 with their bioactive measure.

Compound	Bioactivity Measure	Reported Value	Tested Microbial Strain
		(Units in µM)	
**90**	IC_50_	0.0067 ± 0.0005 µM	*FOX* M15-Pa
**91**		0.23 ± 0.05 µM	
**2**	IC_50_	0.055 µM	*FOXf.* sp *Albedinis*
**3**		0.079 µM	
**4**		0.092 µM	
**104**		0.2 µg mL^−1^ (0.45)	*FOX*
**105**	MIC	0.2 µg mL^−1^ (0.45)	
**106**		0.2 µg mL^−1^ (0.61)	
**107**	MIC	0.312 µg mL^−1^	*FOX* CTM 10402
		(0.77)	
	MFC	2.5 µg mL^−1^	
		(6.16)	
**108**	MIC	0.98 µg mL^−1^ (2.21)	*FOX* ATCC 7601
**109**		1.95 µg mL^−1^ (4.43)	
**110**		1.95 µg mL^−1^ (5.29)	
**40**	MIC	1 µg mL^−1^	*FOX*
		(2.22)	
**41**		1 µg mL^−1^	
		(2.22)	
**39**		1 µg mL^−1^	
		(2.84)	
**92**	IC_50_	2.3 ± 1.0 µM	*FOX* M15-Pa
**93**		15.2 ± 1.4 µM	
**94**		23 ± 8 µM	
**77**	MIC	3.12 µg mL^−1^ (8.68)	*FOX*
**78**		3.12 µg mL^−1^ (8.68)	
**111**	MIC	3.12 µg mL^−1^ (8.73)	*FOX*
**112**		6.25 µg mL^−1^ (17.78)	
**26**	MIC	3.9 µg mL^−1^	*FOX* AB18
		(10.84)	
**27**		7.8 µg mL^−1^	
		(26.46)	
**75**	MIC	8 µg mL^−1^ (12.11)	*FOX* MTCC 2480
**76**		8 µg mL^−1^ (12.11)	
**21**	EC_50_	4.1 µg mL^−1^ (13.04)	*FOXf.* sp*. lycopersici*
**22**		7.4 µg mL^−1^ (25.40)	
**28**	MIC	6.25 µg mL^−1^ (13.28)	*FOX*
**95**	MIC	6.25 µg mL^−1^	*FOX*
		(24.13)	
**96**		6.25 µg mL^−1^	
		(21.83)	
**97**		6.25 µg mL^−1^	
		(20.01)	
**98**		6.25 µg mL^−1^	
		(13.34)	
**99**		12.5 µg mL^−1^	
		(32.51)	
**51**	EC_50_	8.221 µg mL^−1^ (14.58)	*FOX* (S-chl) f.sp
**52**		9.171 µg mL^−1^ (17.48)	
**53**		9.339 µg mL^−1^ (18.36)	
**54**		8.359 µg mL^−1^ (20.82)	
**15**	MIC	16 µg mL^−1^ (16.41)	*FOXf.* sp. *Betae* & *FOX* *f.* sp. *lycopersici*
**16**		16 µg mL^−1^ (16.89)	
**56**	MIC	9 µg mL^−1^	*FOX*
		(16.63)	
**55**		8 µg mL^−1^	
		(18.65)	
**57**		9 µg mL^−1^	
		(20.84)	
**61**	MIC	16 µg mL^−1^ (17.40)	*FOX*
**64**	MIC	6.25 µg mL^−1^	*FOX* ATCC 16417
		(17.44)	
**65**		12.5 µg mL^−1^	
		(42.91)	
**113**	MIC	7.81 µg mL^−1^	*FOX* RCMB 008002
		(17.93)	
**114**		7.81 µg mL^−1^	
		(19.34)	
**66**	MIC	8 µg mL^−1^ (18.08)	*FOX*
**67**		9 µg mL^−1^ (20.34)	
**17**	MIC	6.25 µg mL^−1^ (21.46)	*FOX*
**18**		6.25 µg mL^−1^ (22.26)	
**83**	MIC	12.5 µg mL^−1^	*FOX* NCIM 1332
		(24.58)	
**85**		25 µg mL^−1^	
		(49.16)	
**84**		25 µg mL^−1^	
		(51.59)	
**100**	MIC	12.5 µg mL^−1^	*FOX*
		(25.85)	
**101**		25 µg mL^−1^	
		(54.28)	
**29**	MIC	12.5 µg mL^−1^ (27.69)	*FOX* ATCC 16417
**30**		12.5 µg mL^−1^ (30.61)	
**31**	EC_50_	10.1 µg mL^−1^ (27.79)	*FOX*
**32**		10.6 µg mL^−1^ (29.33)	
**115**	MIC	8 µg mL^−1^ (27.94)	*FOX*
**116**		8 µg mL^−1^ (29.60)	
**86**	MIC	12.5 µg mL^−1^ (28.42)	*FOX* NCIM 1332
**88**	MIC	15.62 µg mL^−1^	*FOX* RCMB 008002
		(28.53)	
**89**		15.62 µg mL^−1^	
		(38.52)	
**87**		15.62 µg mL^−1^	
		(44.86)	
**10**	MIC	15.6 µg mL^−1^	*FOX* RCMB
		(36.24)	8002
**11**		15.6 µg mL^−1^	
		(37.64)	
**12**		15.6 µg mL^−1^	
		(39.15)	
**33**	MIC	12.5 µg mL^−1^ (36.36)	*FOX*
**34**		12.5 µg mL^−1^ (36.83)	
**117**	MIC	12.50 µg mL^−1^ (37.45)	*FOX* ATCC 16417
**118**		12.50 µg mL^−1^ (49.74)	
**119**		12.50 µg mL^−1^ (49.74)	
**102**	MIC	16 ± 1 µg mL^−1^ (37.63)	*FOX*
**103**		18 ± 2 µg mL^−1^ (59.34)	
**7**	MIC	21 ± 3 µg mL^−1^ (39.14)	*FOX*
**8**		23 ± 0 µg mL^−1^ (41.63)	
**23**	MIC	25 µg mL^−1^ (39.84)	*FOX*
**24**		25 µg mL^−1^ (47.40)	
**42**	MIC	21 ± 2 µg mL^−1^	*FOX*
		(42.12)	
**43**		22 ± 2 µg mL^−1^	
		(46.27)	
**44**		20 ± 2 µg mL^−1^	
		(45.99)	
**25**	MIC	25 µg mL^−1^ (44.74)	*FOX*
**81**	MIC	25 µg mL^−1^ (44.75)	*FOX* MTCC 284
**82**		25 µg mL^−1^ (57.01)	
**68**	EC_80_	20 µg mL^−1^ (45.50)	*FOX* NCIM 1332
**69**		20 µg mL^−1^ (47.00)	
**19**	MIC	12.5 µg mL^−1^	*FOX*
		(45.58)	
**20**		12.5 µg mL^−1^	
		(47.40)	
**120**	MIC	16 µg mL^−1^	*FOX* PTCC 5115
		(46.82)	
**5**	IC_50_	52 ± 5 µM	*FOX*
**6**		56 ± 3 µM	
**121**	IC_50_	0.02 mg mL^−1^	*FOX*
		(53.69)	
**9**	MIC	25 µg mL^−1^	*FOX*
		(57.27)	
**13**	MIC	16 µg mL^−1^	*FOX* ATCC 48112
		(58.24)	
**14**		32 µg mL^−1^	
		(96.44)	
**45**	MIC	30 µg mL^−1^ (59.93)	*FOX*
**46**		35 µg mL^−1^ (64.15)	
**47**		30 µg mL^−1^	
		(66.59)	
**48**		35 µg mL^−1^ (72.83)	
**122**	MIC	25 ± 1.443 µg mL^−1^	*FOX*
		(61.14)	
**123**		25 ± 2.500 µg mL^−1^	
		(64.03)	
**1**	MIC	19.8 ± 6.4	*FOX* ATCC 48112
		µg mL^−1^	
		(65.46)	
**71**	MIC	25 µg mL^−1^ (66.45)	*FOX* NCIM 1332
**72**		25 µg mL^−1^ (66.45)	
**49**	EC_50_	18.0 µg mL^−1^ (67.10)	*FOX*
**50**		21.5 µg mL^−1^ (84.56)	
**35**	MIC	25 µg mL^−1^ (70.67)	*FOX* NCIM 1332
**36**		25 µg mL^−1^ (71.56)	
**124**	MIC	25 µg mL^−1^ (71.39)	*FOX*
**125**		25 µg mL^−1^ (79.04)	
**127**	MIC	25 µg mL^−1^	*FOX*
		(76.36)	
**79**	MIC	32 µg mL^−1^	*FOX* NCIM 1008
		(77.19)	
**73**	MIC	30 µg mL^−1^	*FOX* NCIM 1332
		(78.63)	
**74**		40 µg mL^−1^	
		(90.50)	
**37**	MIC	30 µg mL^−1^	*FOX* NCIM 1332
		(78.86)	
**38**		28 µg mL^−1^	
		(92.00)	
**128**	EC_50_	6.25 µg mL^−1^ (80.54)	*FOX*
**129**		6.25 µg mL^−1^ (98.52)	
**130**	MIC_80_	37 µg mL^−1^	*FOX* NCIM 1332
		(84.97)	
**131**		35 µg mL^−1^	
		(86.11)	
**132**		40 µg mL^−1^	
		(98.41)	
**70**	IC_50_	0.086 mM (86.00)	*FOX f.* sp. *albedinis*
**126**	MIC	50 µg mL^−1^	*FOX*
		(87.66)	
**80**	EC_50_	29.89 µg mL^−1^ (98.60)	*FOX*

## Data Availability

Data sharing not applicable. No new data were created or analysed in this study.

## Supplementary Materials

The following are available online, Table S1: Compendium of the most promising organic compounds against several *Fusarium oxysporum* synthesized between 2010–2020 with their bioactive measure.

## Sample Availability

Samples of the compounds are not available from the authors.
