# Supplementary material for: A Compendium of the Most Promising Synthesized Organic Compounds against Several Fusarium oxysporum Species: Synthesis, Antifungal Activity, and Perspectives
Source: Molecules. 2021 Jun 30;26(13):3997. doi: 10.3390/molecules26133997 (PMC8271819; doi:10.3390/molecules26133997)
Supplement: Supplementary file 1 [file molecules-26-03997-s001.zip › molecules-1261352-supplementary.pdf]

Table S1. Compendium of the most promising organic compounds against several Fusarium oxysporum synthesized between 2010-2020 with their bioactive measure.

Compounds		Bioactivity measure	Reported value (Units in µM)	Tested Microbial Strain	Reference
Core	Substituents and label				
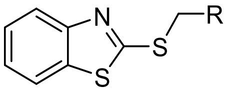	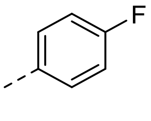 **90**	IC_{50}	0.0067 ± 0.0005 µM	*Fusarium oxysporum* M15-Pa	Ballari, 2017
	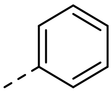 **91**		0.23 ± 0.05 µM		
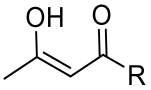	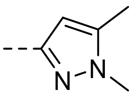 **2**	IC_{50}	0.055 µM	*Fusarium oxysporum* f.sp *Albedinis*	Radi, 2015
	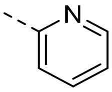 **3**		0.079 µM		
	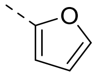 **4**		0.092 µM		
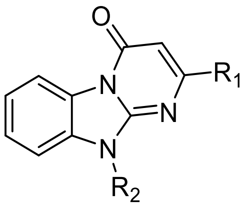	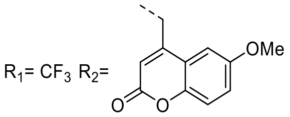 **104**	MIC	0.2 µg mL^{-1} (0.45)	*Fusarium oxysporum*	Puttaraju, 2013
	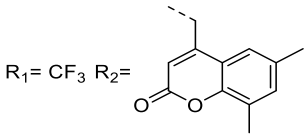 **105**		0.2 µg mL^{-1} (0.45)		
	R_1= $PhCF_3$ R_2= H **106**		0.2 µg mL^{-1} (0.61)		
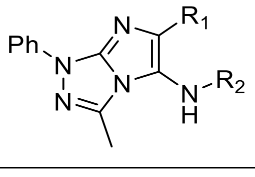	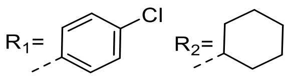 **107**	MIC	0.312 µg mL^{-1} (0.77)	*Fusarium oxysporum* CTM 10402	Aouali, 2015
		MFC	2.5 µg mL^{-1} (6.16)		

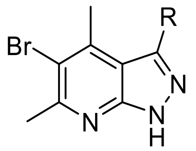	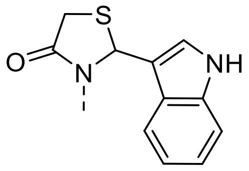 **108**	MIC	0.98 μg mL^{-1} (2.21)	*Fusarium oxysporum* ATCC 7601	Salem, 2016
	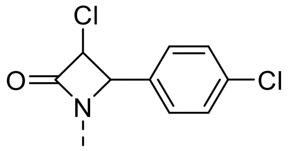 **109**		1.95 μg mL^{-1} (4.43)		
	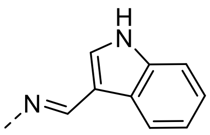 **110**		1.95 μg mL^{-1} (5.29)		
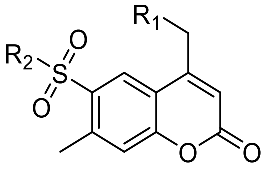	R_1= N_3 R_2= 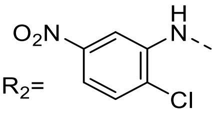 **40**	MIC	1 μg mL^{-1} (2.22)	*Fusarium oxysporum*	Basanagouda, 2010
	R_1= N_3 R_2= 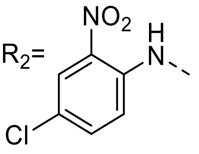 **41**		1 μg mL^{-1} (2.22)		
	R_1=Br R_2= Cl **39**		1 μg mL^{-1} (2.84)		
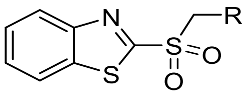	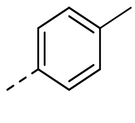 **92**	IC_{50}	2.3 ± 1.0 μM	*Fusarium oxysporum* M15-Pa	Ballari, 2019
	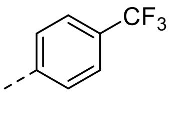 **93**		15.2 ± 1.4 μM		
	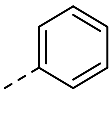 **94**		23 ± 8 μM		
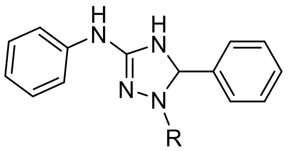	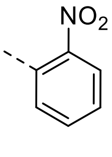 **77**	MIC	3.12 μg mL^{-1} (8.68)	*Fusarium oxysporum*	Yusuf, 2019

	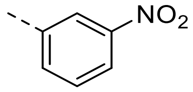 **78**		3.12 μg mL-1 (8.68)		
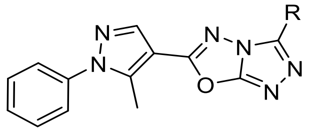	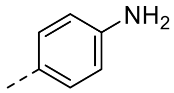 **111**	MIC	3.12 μg mL-1 (8.73)	*Fusarium oxysporum*	Kumar, 2018
	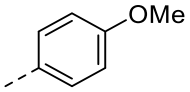 **112**		6.25 μg mL-1 (17.78)		
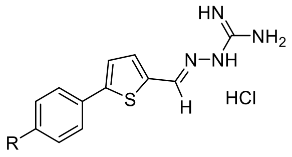	R= Br **26**	MIC	3.9 μg mL-1 (10.84)	*Fusarium oxysporum* AB18	Ajdačić, 2016
	R= CH_3 **27**		7.8 μg mL-1 (26.46)		
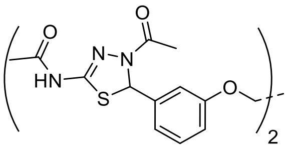	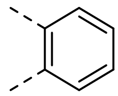 **75**	MIC	8 μg mL-1 (12.11)	*Fusarium oxysporum* MTCC 2480	Yusuf, 2014
	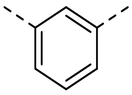 **76**		8 μg mL-1 (12.11)		
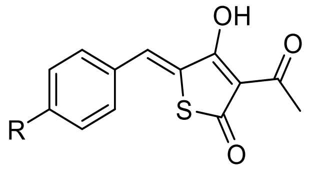	R=CF_3 **21**	EC_{50}	4.1 μg mL-1 (13.04)	*Fusarium oxysporum* f. sp. *lycopersici*	Lv, 2018
	R=NO_2 **22**		7.4 μg mL-1 (25.40)		
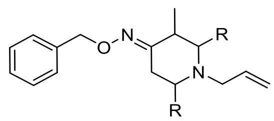	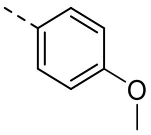 **28**	MIC	6.25 μg mL-1 (13.28)	*Fusarium oxysporum*	Narayanan, 2012
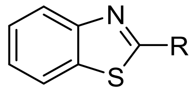	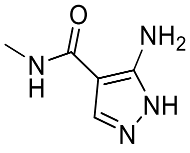 **95**	MIC	6.25 μg mL-1 (24.13)	*Fusarium oxysporum*	Bondock, 2009 and 2010

	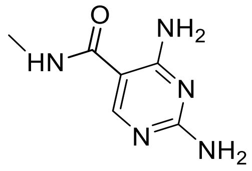<br>**96**		6.25 μg mL^{-1}<br>(21.83)		
	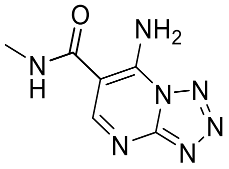<br>**97**		6.25 μg mL^{-1}<br>(20.01)		
	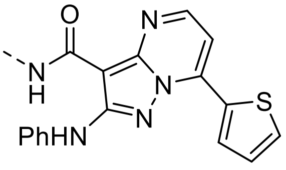<br>**98**		6.25 μg mL^{-1}<br>(13.34)		
	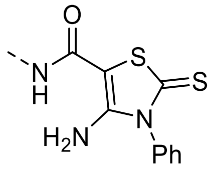<br>**99**		12.5 μg mL^{-1}<br>(32.51)		
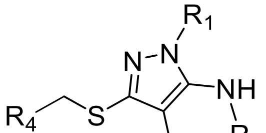	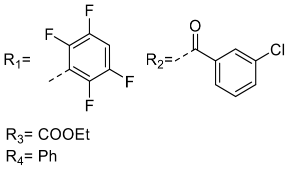<br>**51**	EC50	8.221 μg mL^{-1}<br>(14.58)	*Fusarium oxysporum* (S-chl) f.sp	Liu, 2014
	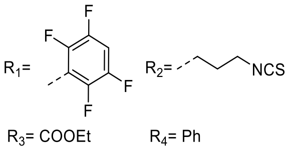<br>**52**		9.171 μg mL^{-1}<br>(17.48)		
	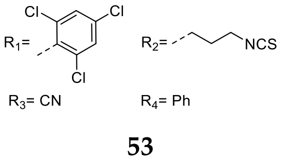<br>**53**		9.339 μg mL^{-1}<br>(18.36)		
	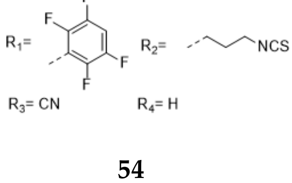<br>**54**		8.359 μg mL^{-1}<br>(20.82)		

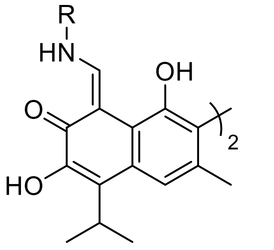	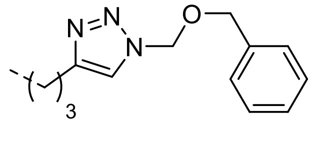 **15**	MIC	16 µg mL^{-1} (16.41)	*Fusarium oxysporum* f. sp. *Betae* & *Fusarium oxysporum* f. sp. *lycopersici*	Pyta, 2016
	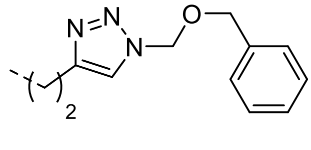 **16**		16 µg mL^{-1} (16.89)		
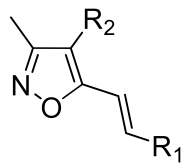	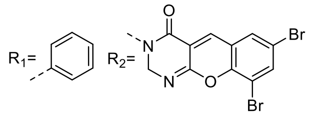 **56**	MIC	9 µg mL^{-1} (16.63)	*Fusarium oxysporum*	Rajanarendar, 2012
	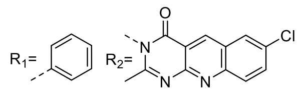 **55**		8 µg mL^{-1} (18.65)		
	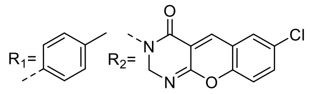 **57**		9 µg mL^{-1} (20.84)		
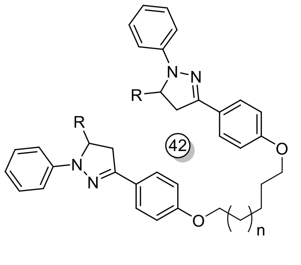	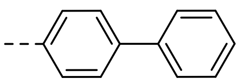 n: 8 **61**	MIC	16 µg mL^{-1} (17.40)	*Fusarium oxysporum*	Yusuf, 2019
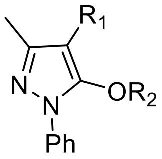	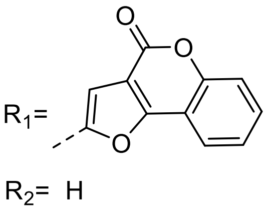 **64**	MIC	6.25 µg mL^{-1} (17.44)	*Fusarium oxysporum* ATCC 16417	Bondock, 2011
	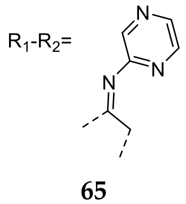 **65**		12.5 µg mL^{-1} (42.91)		

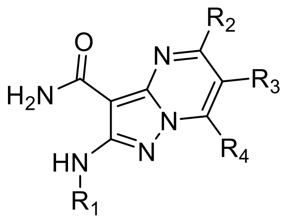	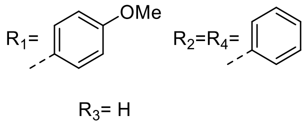 **113**	MIC	7.81 μg mL^{-1} (17.93)	*Fusarium oxysporum* RCMB 008002	Hassan, 2017
	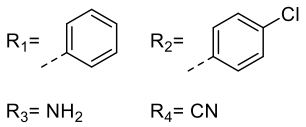 **114**		7.81 μg mL^{-1} (19.34)		
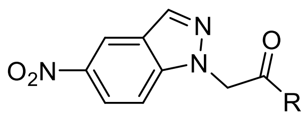	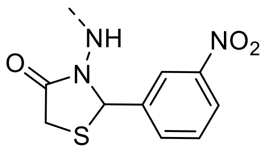 **66**	MIC	8 μg mL^{-1} (18.08)	*Fusarium oxysporum*	Upadhyay, 2010
	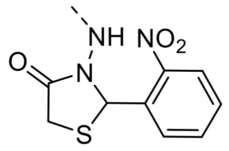 **67**		9 μg mL^{-1} (20.34)		
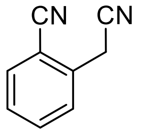	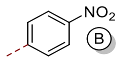 **17**	MIC	6.25 μg mL^{-1} (21.46)	*Fusarium oxysporum*	Fadda, 2013
	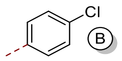 **18**		6.25 μg mL^{-1} (22.26)		
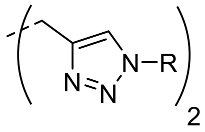	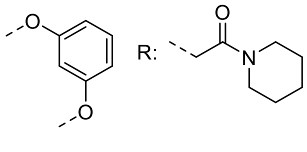 **83**	MIC	12.5 μg mL^{-1} (24.58)	*Fusarium oxysporum* NCIM 1332	Deshmukh, 2019
	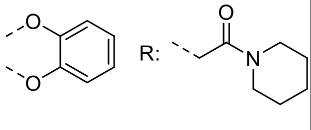 **85**		25 μg mL^{-1} (49.16)		
	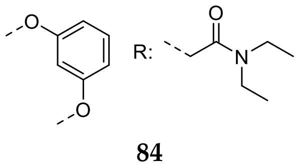 **84**		25 μg mL^{-1} (51.59)		
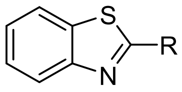	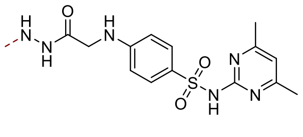 **100**	MIC	12.5 μg mL^{-1} (25.85)	*Fusarium oxysporum*	Fadda, 2019

	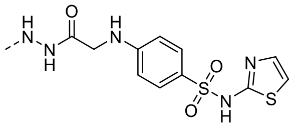 **101**		25 μg mL^{-1} (54.28)		
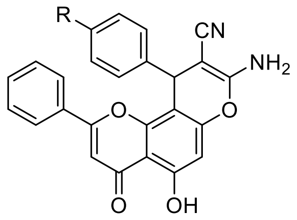	R: NMe_2 **29**	MIC	12.5 μg mL^{-1} (27.69)	*Fusarium oxysporum* ATCC 16417	Ramesh, 2015
	R: H **30**		12.5 μg mL^{-1} (30.61)		
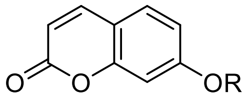	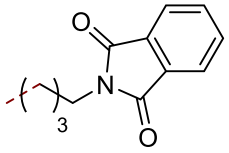 **31**	EC_{50}	10.1 μg mL^{-1} (27.79)	*Fusarium oxysporum*	Pan, 2017
	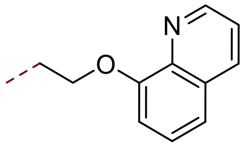 **32**		10.6 μg mL^{-1} (29.33)		
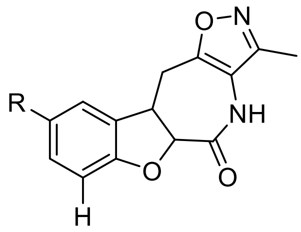	R: OCH_3 **115**	MIC	8 μg mL^{-1} (27.94)	*Fusarium oxysporum*	Rajanarendar, 2013
	R: CH_3 **116**		8 μg mL^{-1} (29.60)		
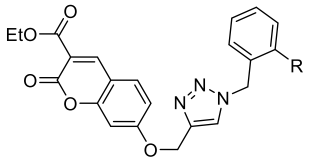	R: Cl **86**	MIC	12.5 μg mL^{-1} (28.42)	*Fusarium oxysporum* NCIM 1332	Shaikh, 2015
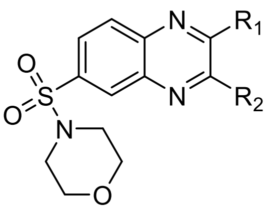	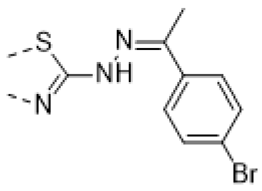 **88**	MIC	15.62 μg mL^{-1} (28.53)	*Fusarium oxysporum* RCMB 008002	Ammar, 2020

	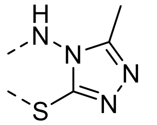 **89**		15.62 μg mL^{-1} (38.52)		
	R_1, R_2 = Cl **87**		15.62 μg mL^{-1} (44.86)		
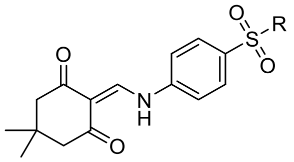	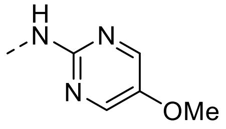 **10**	MIC	15.6 μg mL^{-1} (36.24)	*Fusarium oxysporum* RCMB 008002	Ghorab, 2017
	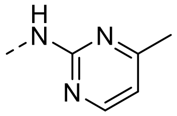 **11**		15.6 μg mL^{-1} (37.64)		
	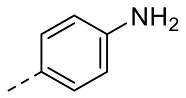 **12**		15.6 μg mL^{-1} (39.15)		
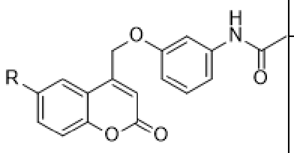	R: Cl **33**	MIC	12.5 μg mL^{-1} (36.36)	*Fusarium oxysporum*	Makandar, 2012
	R: OMe **34**		12.5 μg mL^{-1} (36.83)		
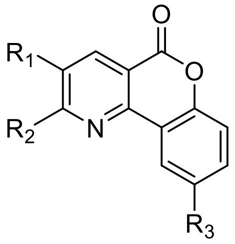	R_1, R_2= 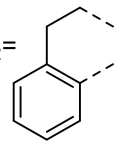 R_3 : Cl **117**	MIC	12.50 μg mL^{-1} (37.45)	*Fusarium oxysporum* ATCC 16417	Patel, 2012
	R_1, R_2= 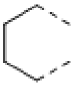 R_3= H **118**		12.50 μg mL^{-1} (49.74)		
	R_1, R_2= 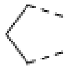 R_3= CH_3 **119**		12.50 μg mL^{-1} (49.74)		
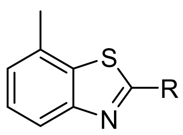	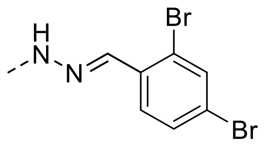 **102**	MIC	16 ± 1 μg mL^{-1} (37.63)	*Fusarium oxysporum*	Zha, 2017

	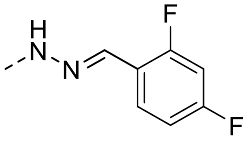<br>**103**		18 ± 2 μg mL^{-1} (59.34)		
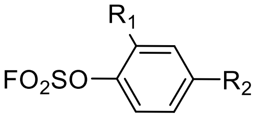	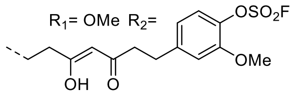<br>**7**	MIC	21 ± 3 μg mL^{-1} (39.14)	*Fusarium oxysporum*	Ravindar, 2018
	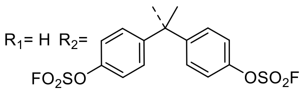<br>**8**		23 ± 0 μg mL^{-1} (41.63)		
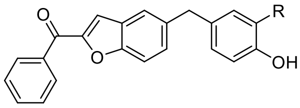	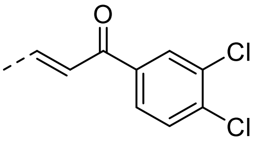<br>**23**	MIC	25 μg mL^{-1} (39.84)	*Fusarium oxysporum*	Shankar, 2016
	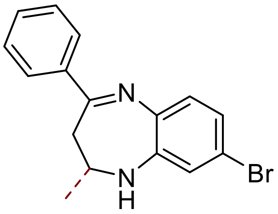<br>**24**		25 μg mL^{-1} (47.40)		
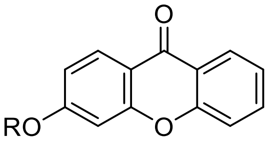	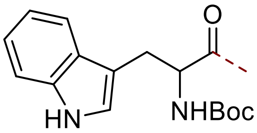<br>**42**	MIC	21 ± 2 μg mL^{-1} (42.12)	*Fusarium oxysporum*	Chen, 2017
	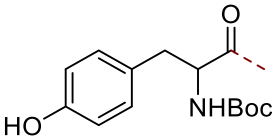<br>**43**		22 ± 2 μg mL^{-1} (46.27)		
	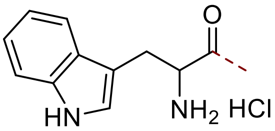<br>**44**		20 ± 2 μg mL^{-1} (45.99)		
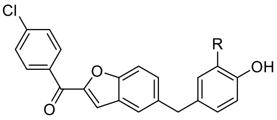	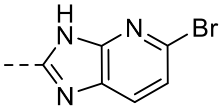<br>**25**	MIC	25 μg mL^{-1} (44.74)	*Fusarium oxysporum*	Shankar, 2018

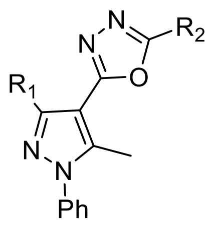	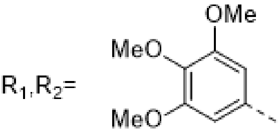 **81**	MIC	25 μg mL-1 (44.75)	*Fusarium oxysporum* MTCC 284	Ningaiah, 2013
	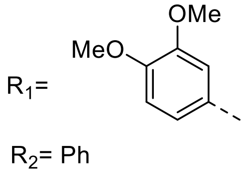 **82**		25 μg mL-1 (57.01)		
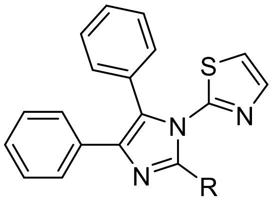	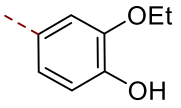 **68**	EC_{80}	20 μg mL-1 (45.50)	*Fusarium oxysporum* NCIM 1332	Nikalje, 2017
	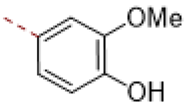 **69**		20 μg mL-1 (47.00)		
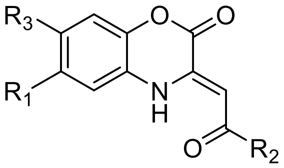	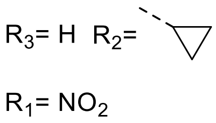 **19**	MIC	12.5 μg mL-1 (45.58)	*Fusarium oxysporum*	Sharma, 2017
	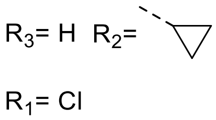 **20**		12.5 μg mL-1 (47.40)		
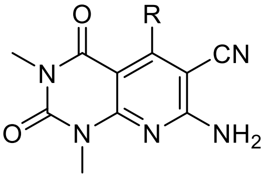	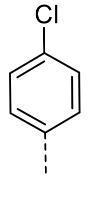 **120**	MIC	16 μg mL-1 (46.82)	*Fusarium oxysporum* PTCC 5115	Aryan, 2018
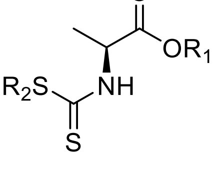	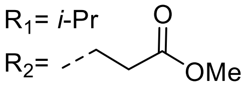 **5**	IC_{50}	52 ± 5 μM	*Fusarium oxysporum*	Quiroga, 2019
	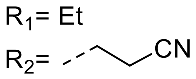 **6**		56 ± 3 μM		

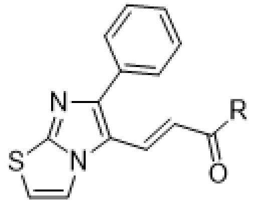	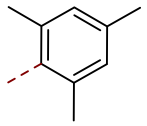 **121**	IC50	0.02 mg mL-1 (53.69)	*Fusarium oxysporum*	Koudad, 2019
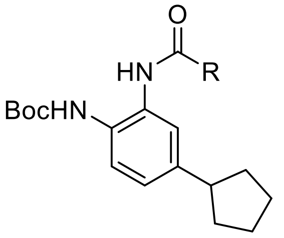	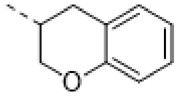 **9**	MIC	25 μg mL-1 (57.27)	*Fusarium oxysporum*	Pochampally, 2014
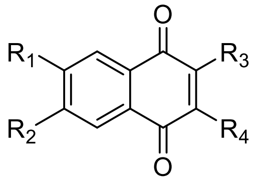	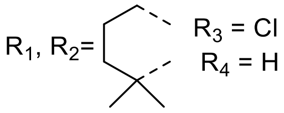 **13**	MIC	16 μg mL-1 (58.24)	*Fusarium oxysporum* ATCC 48112	Castro, 2013
	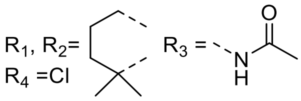 **14**		32 μg mL-1 (96.44)		
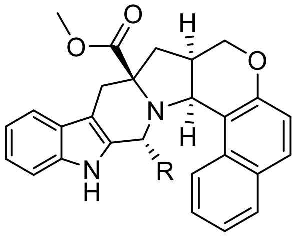	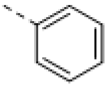 **45**	MIC	30 μg mL-1 (59.93)	*Fusarium oxysporum*	Arumugam, 2012
	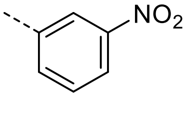 **46**		35 μg mL-1 (64.15)		
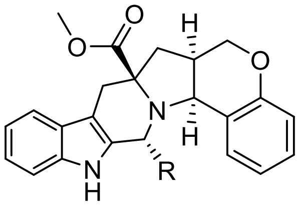	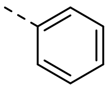 **47**		30 μg mL-1 (66.59)		
	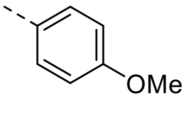 **48**		35 μg mL-1 (72.83)		
	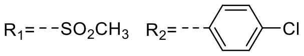 **122**	MIC	25 ± 1.443 μg mL-1 (61.14)	*Fusarium oxysporum*	Sangshetti, 2011

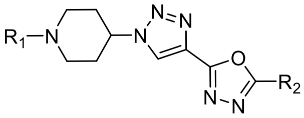	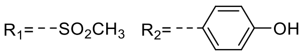 **123**		25 ± 2.500 μg mL^{-1} (64.03)		
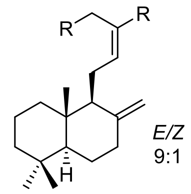	R= CHO **1**	MIC	19.8 ± 6.4 μg mL^{-1} (65.46)	*Fusarium oxysporum* ATCC 48112	Gonzalez, 2010
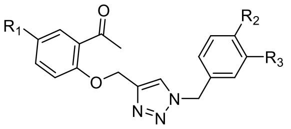	R_1= Cl R_2= Cl R_3= H **71**	MIC	25 μg mL^{-1} (66.45)	*Fusarium oxysporum* NCIM 1332	Shaikh, 2016
	R_1= Cl R_2= H R_3= Cl **72**		25 μg mL^{-1} (66.45)		
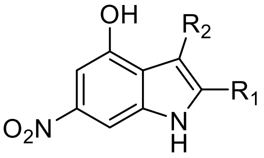	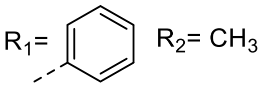 **49**	EC_{50}	18.0 μg mL^{-1} (67.10)	*Fusarium oxysporum*	Kokurkina, 2011
	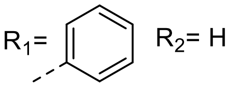 **50**		21.5 μg mL^{-1} (84.56)		
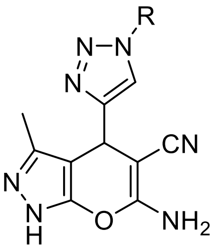	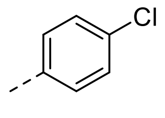 **35**	MIC	25 μg mL^{-1} (70.67)	*Fusarium oxysporum* NCIM 1332	Khare, 2019
	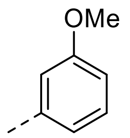 **36**		25 μg mL^{-1} (71.56)		
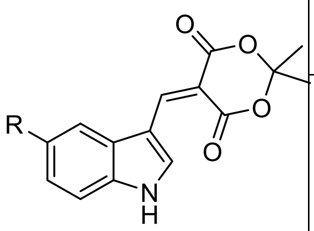	R= Br **124**	MIC	25 μg mL^{-1} (71.39)	*Fusarium oxysporum*	Thirupathi, 2014
	R= NO_2 **125**		25 μg mL^{-1} (79.04)		
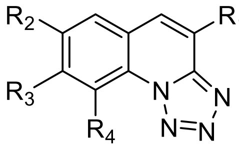	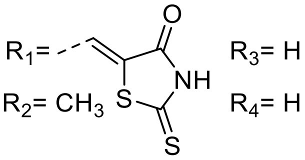	MIC	25 μg mL^{-1} (76.36)	*Fusarium oxysporum*	Subhedar, 2016

	**127**				
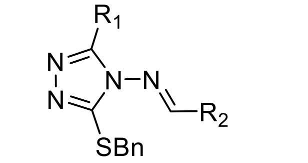	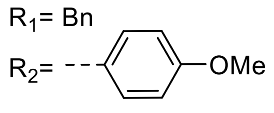<br>**79**	MIC	32 µg mL^{-1}<br>(77.19)	*Fusarium oxysporum*<br>NCIM 1008	Bathia, 2011
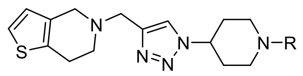	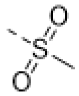<br>**73**	MIC	30 µg mL^{-1}<br>(78.63)	*Fusarium oxysporum*<br>NCIM 1332	Darandale, 2013
	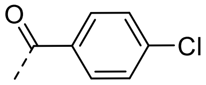<br>**74**		40 µg mL^{-1}<br>(90.50)		
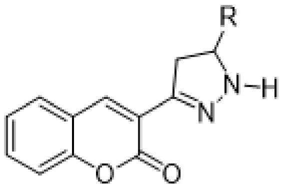	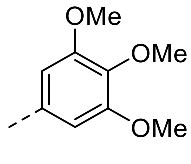<br>**37**	MIC	30 µg mL^{-1}<br>(78.86)	*Fusarium oxysporum*<br>NCIM 1332	Chate, 2019
	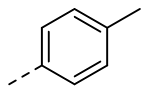<br>**38**		28 µg mL^{-1}<br>(92.00)		
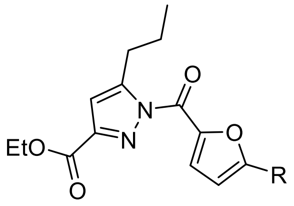	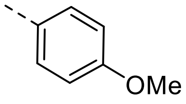<br>**128**	EC_{50}	6.25 µg mL^{-1}<br>(80.54)	*Fusarium oxysporum*	Ahmed, 2019
	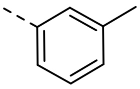<br>**129**		6.25 µg mL^{-1}<br>(98.52)		
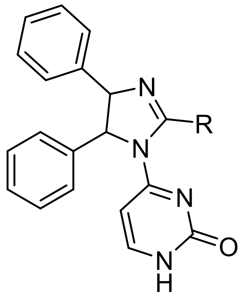	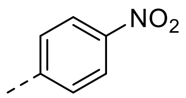<br>**130**	MIC_{80}	37 µg mL^{-1}<br>(84.97)	*Fusarium oxysporum*<br>NCIM 1332	Tiwari, 2018
	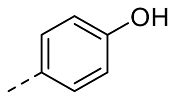<br>**131**		35 µg mL^{-1}<br>(86.11)		
	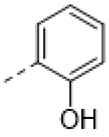		40 µg mL^{-1}<br>(98.41)		

	**132**				
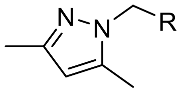	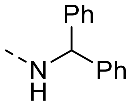 **70**	IC_{50}	0.086 mM (86.00)	*Fusarium oxysporum* f.sp. *albedinis*	Abrigach, 2017
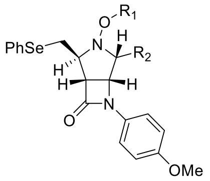	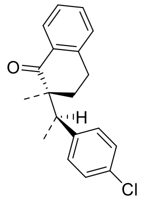 **126**	MIC	50 µg mL^{-1} (87.66)	*Fusarium oxysporum*	Arumugam, 2010
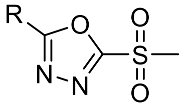	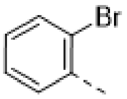 **80**	EC_{50}	29.89 µg mL^{-1} (98.60)	*Fusarium oxysporum*	Xu, 2011
